# Simultaneous Photocatalytic Production of H_2_ and Acetal from Ethanol with Quantum Efficiency over 73% by Protonated Poly(heptazine imide) under Visible Light

**DOI:** 10.1021/acscatal.4c04180

**Published:** 2024-09-23

**Authors:** Vitaliy Shvalagin, Nadezda Tarakina, Bolortuya Badamdorj, Inga-Marie Lahrsen, Eleonora Bargiacchi, Andre Bardow, Ziqi Deng, Wenchao Wang, David Lee Phillips, Zhengxiao Guo, Guigang Zhang, Junwang Tang, Oleksandr Savateev

**Affiliations:** † Max Planck Institute of Colloids and Interfaces, Am Mühlenberg 1, Potsdam 14476, Germany; ‡ Energy & Process Systems Engineering, Department of Mechanical and Process Engineering, ETH Zurich, Tannenstrasse 3, Zurich 8092, Switzerland; § Department of Chemistry, The University of Hong Kong, Kowloon, Hong Kong SAR 999077, China; ∥ State Key Laboratory of Photocatalysis on Energy and Environment, College of Chemistry, Fuzhou University, Fujian 350116, China; ⊥ Industrial Catalysis Center, Department of Chemical Engineering, Tsinghua University, Beijing 100084, China; # Department of Chemistry, The Chinese University of Hong Kong, Shatin, New Territories, Kowloon 999077, Hong Kong

**Keywords:** protonated poly(heptazine imide), salt melt treatment, nanostructure, photocatalysis, hydrogen evolution, organic synthesis, visible light

## Abstract

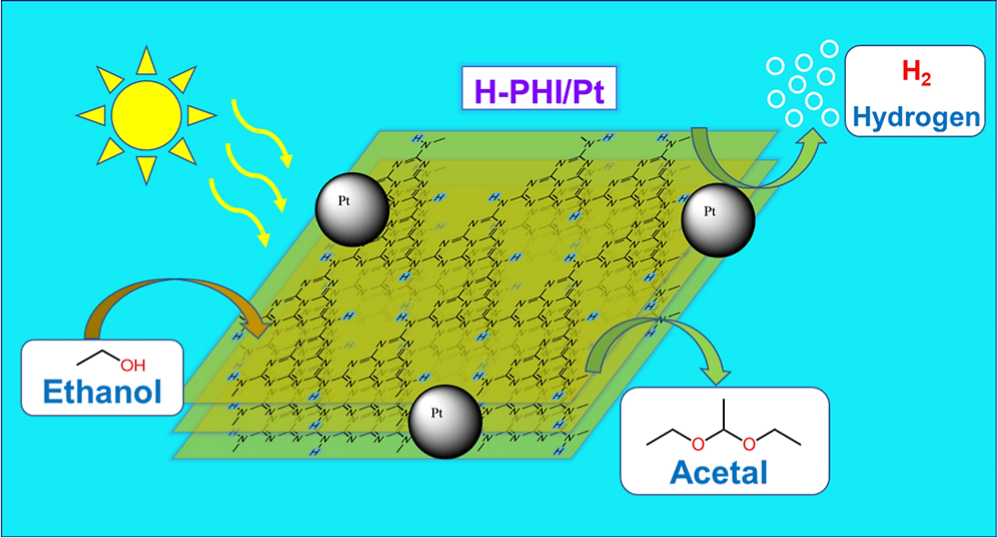

In this work, protonated poly(heptazine imide) (H-PHI) was obtained by adding acid to the suspension of potassium PHI (K-PHI) in ethanol. It was established that the obtained H-PHI demonstrates very high photocatalytic activity in the reaction of hydrogen formation from ethanol in the presence of Pt nanoparticles under visible light irradiation in comparison with K-PHI. This enhancement can be attributed to improved efficiency of photogenerated charge transfer to the photocatalyst’s surface, where redox processes occur. Various factors influencing the system’s activity were evaluated. Notably, it was discovered that the conditions of acid introduction into the system can significantly affect the size of Pt (cocatalyst metal) deposition on the H-PHI surface, thereby enhancing the photocatalytic system’s stability in producing molecular hydrogen. It was established that the system can operate efficiently in the presence of air without additional components on the photocatalyst surface to block air access. Under optimal conditions, the apparent quantum yield of molecular hydrogen production at 410 nm is around 73%, the highest reported value for carbon nitride materials to date. The addition of acid not only increases the activity of the reduction part of the system but also leads to the formation of a value-added product from ethanol–1,1-diethoxyethane (acetal) with high selectivity.

## 1 Introduction

The relevance of developing effective methods for obtaining molecular hydrogen has been increasing for a long time.^1−3^ This is caused both by the critical pollution of the environment by hydrocarbon fuel combustion products, which prompts humanity to switch to environmentally friendly hydrogen fuel, and by the development and improvement of new directions for the use of molecular hydrogen. In particular, active use in the automotive industry, as well as in highly efficient hydrogen fuel cells.^4^ Today, the most common methods of hydrogen synthesis are high-temperature conversion of natural gas and other types of hydrocarbon raw materials,^1,2^ as well as water electrolysis.^5^ However, these methods are energy- and resource-consuming, which stimulates researchers to develop other methods for obtaining hydrogen, in particular, using renewable energy sources. One such source can be the inexhaustible light energy of the sun.

Photocatalytic processes involving semiconductor materials occupy an important place among molecular hydrogen synthesis methods that use sunlight energy.^1^ In general, photocatalytic production of hydrogen is represented by two approaches: (i) overall water splitting^6,7^ and (ii) photocatalytic production of hydrogen from aqueous solutions of “sacrificial” compounds—electron donors.^8−10^ The main disadvantages of these approaches, which limit their practical application, are (i) the low efficiency of the photocatalytic decomposition of water under visible light irradiation,^11^ (ii) the use of toxic and expensive compounds for obtaining photocatalysts and cocatalysts,^12^ (iii) the formation of explosive gas mixtures of hydrogen and oxygen,^11,13^ and (iv) the need to separate them for further use.^13^ Also, in these cases, half of the photogenerated charges, namely, valence band holes, are spent to obtain molecular oxygen, which has low value, or oxidize additional reagents, electron donors, and produce waste.

The most promising is the simultaneous use of both types of photogenerated charges.^14^ Namely, photogenerated electrons are employed to reduce water to molecular hydrogen. At the same time, the photogenerated holes are used to oxidize the electron donor to value-added products. This approach not only increases the efficiency of photocatalytic hydrogen production, eliminates the formation of explosive gas mixtures (hydrogen–oxygen) and the need for their separation, but also yields valuable oxidation products of electron donors, the cost of which can exceed many times the cost of hydrogen. These features can make this approach more economically attractive.^15,16^

One promising material for the photocatalytic production of hydrogen from solutions of electron donors is potassium poly(heptazine imide) (K-PHI).^8−10,17−19^ Such material has improved absorption of visible light, a lower number of defects—recombination centers of photogenerated charges, improved crystallinity, and a larger surface area, which leads to a significant increase of its photocatalytic activity in the processes of gas-phase destruction of organic pollutants,^20^ photoredox catalytic organic transformations,^21^ CO_2_ reduction,^22^ hydrogen evolution from aqueous solutions of electron donors,^8−10,17,18,22^ etc.

In this work, it was established that the addition of a small amount of acid to K-PHI suspensions leads to its in situ conversion into protonated PHI (H-PHI), which has a very high photocatalytic activity in the reaction of simultaneous production of hydrogen from ethanol solutions and the formation of 1,1-diethoxyethane (acetal) in solution under visible light irradiation. Additionally, in the presence of acid, we significantly reduced the content of the metal cocatalyst (Pt), which is essential for the dark processes in photocatalytic hydrogen production systems.^18,23^ Among the high-value chemicals that can be obtained by the oxidation of ethanol, 1,1-diethoxyethane is one of the most important because it can be used in the perfumery and pharmaceutical industries, as a starting product for organic synthesis, as a solvent, as an additive to diesel fuel, etc.^24^ The direct photocatalytic conversion of ethanol into acetal mediated by PHI material has been shown for the first time.

## 2 Materials and Methods

### 2.1 Materials

Commercially available melamine, KCl, LiCl, K_2_PtCl_4_, HCl, ethanol, and acetal were used without further purification.

### 2.2 Synthesis

Bulk g-C_3_N_4_ was obtained by the thermal treatment of melamine at 550 °C in the presence of air. In a typical procedure, melamine (10 g) was placed in a porcelain crucible and placed in a tubular oven. The oven was heated at a rate of 2.3 °C min^–1^ from 25 to 550 °C. The mixture was kept at 550 °C for 4 h, after which it was allowed to cool to room temperature spontaneously. The resulting yellow powder was ground in a ball mill for 5 min and used for further research.

K-PHI was obtained by additional thermal treatment of bulk g-C_3_N_4_ in the melt of the eutectic mixture KCl/LiCl. In a typical procedure, g-C_3_N_4_ (1 g), KCl (5.5 g), and LiCl (4.5 g) were ground in a planetary ball mill for 5 min, after which the mixture was placed in a tubular oven. The oven was purged with argon at the constant flow 1 L min^–1^ and heated at a rate of 2.3 °C min^–1^ from 25 to 550 °C. The mixture was kept at 550 °C for 4 h, after which it was allowed to cool to room temperature spontaneously. The obtained material was washed several times with distilled water to remove salt residues and finally with ethanol. After each washing step, the suspension was centrifuged at 4000 rpm to separate solid material from solution. Then the sample was dried at 60 °C in the presence of air. The resulting yellow powder was collected without grounding and used for further research.

H-PHI was obtained by an additional treatment of K-PHI in HCl solution. In a typical procedure, K-PHI (100 mg) was placed in HCl solution (40 mL, 0.1 M). The mixture was stirred for 2 h on a magnetic stirrer at room temperature. The obtained material was washed several times with distilled water to remove acid and finally with ethanol. After each washing step, the suspension was centrifuged at 4000 rpm to separate solid material from solution. Then the sample was dried at 60 °C in the presence of air. The resulting gray powder was collected without grounding and used for further research.

H-PHI/Pt was obtained by irradiation of a deaerated water–ethanol suspension of K-PHI in the presence of K_2_PtCl_4_ and HCl. In a typical procedure, K-PHI (100 mg) was placed in a water–ethanol solution (39.4 mL, 96 wt % ethanol) containing K_2_PtCl_4_ (1.06 mL, 1 mg mL^–1^). The mixture was sealed in a glass reactor and deaerated by purging with argon for 20 min. The reactor was irradiated for 30 min with visible light (λ = 410 nm) while stirring on a magnetic stirrer. Next, HCl (0.4 mL, 1 M) was injected into the reactor using a syringe, and the irradiation was continued for another 2 h. The obtained material was washed several times with distilled water to remove acid and finally with ethanol. After each washing step, the suspension was centrifuged at 4000 rpm to separate the solid material from the solution. Then the sample was dried at 60 °C in the presence of air. The resulting gray powder was collected without grounding and used for further research.

### 2.3 Characterizations

X-ray powder diffraction (XRD) patterns were recorded on a Rigaku SmartLab X-ray diffractometer using Cu Kα_1_ radiation. Fourier-transform infrared (FT-IR) spectra were recorded on a Thermo Scientific Nicolet iD5 spectrometer equipped with an attenuated total reflection unit applying a resolution of 2 cm^–1^. UV–vis spectra were measured on a Shimadzu UV-2600 spectrophotometer equipped with an integrating sphere. PL spectra were recorded on an FP-8300 spectrofluorometer. For transmission electron microscopy (TEM) observations, a suspension of the sample in ethanol was sonicated for 10 min and then drop-casted to a Cu grid with a lacey carbon support and dried for 20 min. TEM and STEM characterizations were performed using a JEOL JEM F200 microscope and a double Cs corrected JEOL JEM-ARM200F microscope operated at 80 kV and equipped with a cold-field emission gun. Elemental combustion analysis was performed using a Vario Micro device. Inductively coupled plasma–optical emission spectrometry (ICP–OES) was conducted by using a HORIBA Ultra 2 instrument equipped with photomultiplier tube detection. The surface area of the samples was examined by the low-temperature N_2_ adsorption–desorption method with a Quantachrome Quadrasorb SI porosimeter and calculated by Brunauer–Emmett–Teller model. Electron paramagnetic resonance (EPR) spectra were performed on an ESR spectrometer (EMX-nano, Bruker). For EPR analysis, the catalyst (10 mg) was dispersed in a solution of ethanol/water (1 mL, 96:4 wt %) using ultrasonic treatment for 10 min. The dispersion of the photocatalyst was transferred into a capillary, the capillary was placed inside the EPR spectrometer, and the spectra were measured (EPR spectra without irradiation). After the measurements, the capillary with the photocatalyst suspension was irradiated with visible light for 5 min (λ = 410 nm), and the EPR spectrum was acquired again (EPR spectra after irradiation). For femtosecond transient absorption (fs-TA) and transient photoluminescence decay measurements, the samples were prepared as follows. Ten mL acetonitrile or ethanol was added to 1 mg sample of g-C_3_N_4_, K-PHI, H-PHI, and H-PHI/Pt and sonicated for 3 h. Subsequently, 0.1 mL of saturated HCl solution was added to all of the samples. This corresponds to a 0.1 M concentration of HCl in the analyzed suspensions. To reveal the influence of the acidic environment on the excited state dynamics, one sample was prepared by dispersing H-PHI/Pt in ethanol without adding HCl. The fs-TA measurements were conducted using the Helios pump–probe transient absorption spectrometer system (Ultrafast Systems, USA) with a fs laser from the Spitfire Pro regenerative amplified Ti:sapphire laser system (Spectra Physics, USA). The 800 nm laser light with 120 fs pulse width was subsequently split into two beams, one as the pump beam and another one as the probe beam. The pump beam passed through a harmonic resonator to generate the 400 nm pump beam (the third harmonic of the fundamental 800 nm), whereas the probe beam passed through a sapphire crystal and generated a white-light continuum (400–850 nm). The time-delayed probe beam was controlled by the optical delay rail, with a maximum temporal delay at 3.3 ns. It would pass through the samples, and the signals were then collected by the detector. A reference probe beam was also used to optimize the signal-to-noise ratio. The samples were measured in suspension in acetonitrile and ethanol with a 2 mm path-length quartz cuvette. The spectrometric data were recorded in a 3D wavelength-time-absorbance matrix.

The subtraction of background, the subtraction of scattering light, and chirp correction were done for all of the data before analysis. The single-wavelength kinetic was extracted from the recorded 3D matrices, and fitting was done by the Surface Explorer V4.5 based on eq 1. The kinetics were selected at the maxima of the positive signal or the minima of the negative signal with the best quality
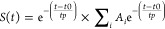
1

The transient photoluminescence decay spectroscopy measurements were based on the FLS 1000 photoluminescence spectrometer (Edinburgh, UK) using the time-correlated single photon counting technique with an EPL-375 nm picosecond pulsed diode laser. The samples were measured in a suspension solution in acetonitrile in a 10 mm path-length quartz cuvette.

### 2.4 Photocatalytic Reactions

The photocatalytic activity of the obtained material was studied by converting ethanol into hydrogen and 1,1-diethoxyethane simultaneously under visible light irradiation. In a typical procedure, a photocatalyst (10 mg) and K_2_PtCl_4_ (0.106 mL, concentration 1 mg mL^–1^, corresponds to 0.5 wt % loading of metallic Pt(0) in the photocatalyst) were added to an aqueous ethanol solution (5 mL, 96 wt % ethanol) and placed into the vial made of borosilicate glass (WHEATON SAMPULE Vial, Glass, 13 mm, 6 mL). Then the vial was sealed using a cap equipped with a silicon/PTFE 3.2 mm septum. After this, the vial was saturated with argon for 10 min and irradiated with visible light while stirring. In this work, a blue LED module (emission maximum λ = 410 nm, measured optical power 85 mW cm^–2^) was used. LEDs with emission maxima at λ = 365 and 465 nm were also used to determine apparent quantum yield (AQY). The intensity of the LED modules was measured using the PM400 Power Meter Console equipped with an S142C Integrating Sphere Photodiode Power Sensor (Si, 350–1100 nm) purchased from Thorlabs. During the irradiation, the temperature of the reaction medium was maintained at about 28 °C using an air fan. During irradiation, to change the acid concentration, 0.05 or 0.5 mL of 1 M HCl was injected into a 6 mL reactor with a syringe to obtain a solution of 0.01 or 0.1 M acid. The volume of hydrogen was measured using a connected series high-performance mass flow controller for gases (Bronkhorst EL-FLOW Prestige FG-201CV) and a water-filled inverted buret equipped with the silicon/PTFE 3.2 mm septum for sampling. To prevent ethanol vapor from going into the mass flow controller, a trap cooled with water was placed between the controller and the photoreactor. The scheme and picture of our photocatalytic system are shown in Figure S1. The concentration of the reaction product was measured using a GCMS Agilent 8890 equipped with HP-5 ms Ultra Inert Columns. To confirm the chemical structure of the product and quantify the chemical composition of the liquid phase, ^1^H and ^13^C NMR spectra were acquired using Ascend 400 MHz NMR spectrometer (Bruker) in acetonitrile-*d*_3_ using 1,3,5-trimethoxybenzene as an internal standard.

AQY of hydrogen is calculated according to the eq 2
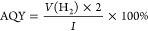
2where *V*(H_2_)—the rate of hydrogen evolution (mol h^–1^) and *I*—the intensity of light (mole photons h^–1^).

To investigate the stability of the photocatalysts, g-C_3_N_4_, K-PHI, and H-PHI, the production of H_2_ under visible light irradiation was monitored in several cycles lasting 120 min each. For this, after in situ deposition of platinum, each of the photocatalysts was irradiated for 120 min. After that, the photocatalyst was washed with ethanol and separated from the solution by centrifugation. Next, a new portion of ethanol containing the required amount of acid (or without acid) was added to the photocatalyst and redispersed in this fresh solvent. Then, the photocatalyst suspension was placed in the reactor, sealed, purged with argon, and irradiated again. This procedure was repeated 3 times for each of the studied photocatalysts.

## 3 Results

### 3.1 Characterization

**Figure 1:**
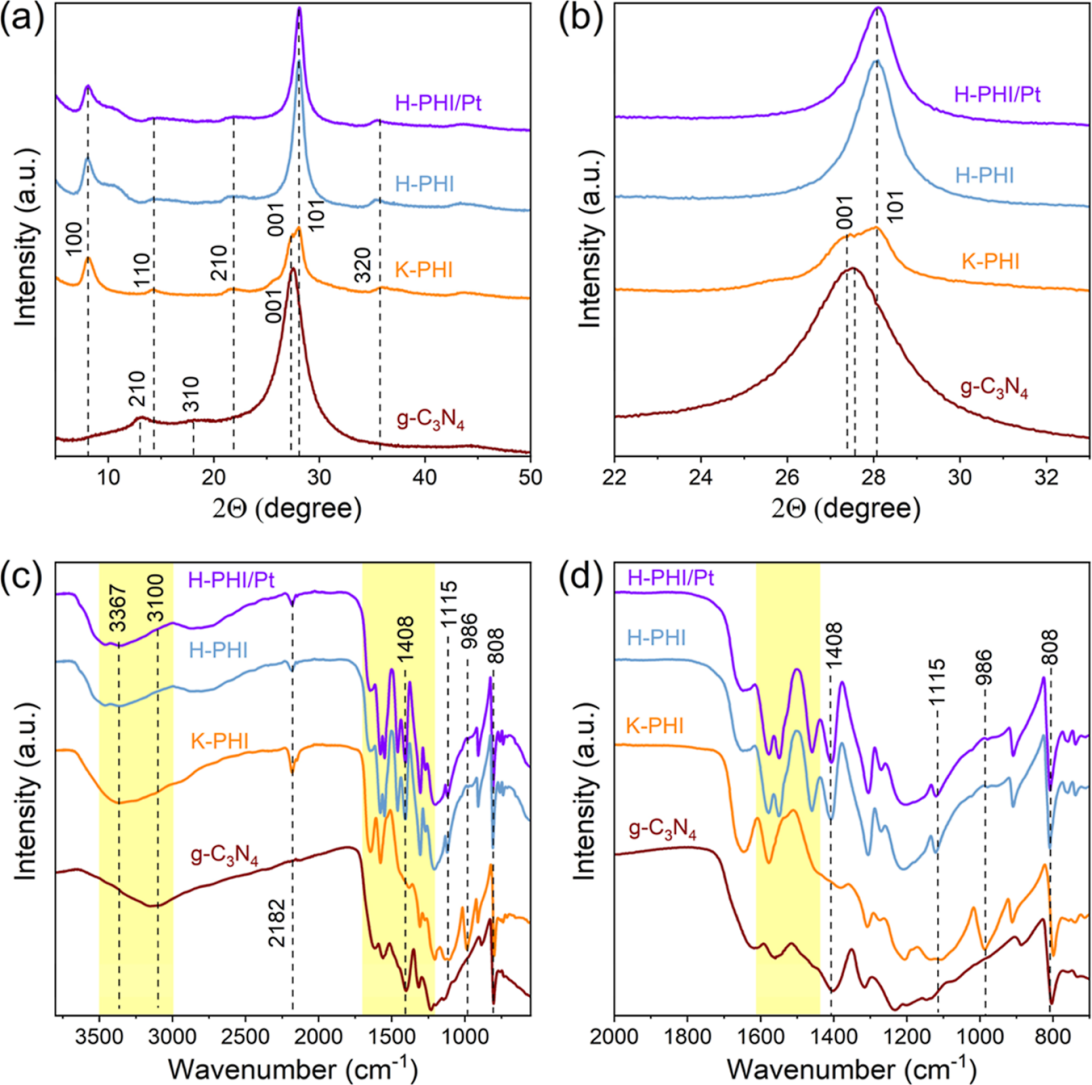
(a,b) XRD patterns and (c,d) FT-IR spectra of g-C_3_N_4_, K-PHI, H-PHI, and H-PHI/Pt.

In the diffraction pattern of bulk carbon nitride (g-C_3_N_4_), obtained by annealing melamine in the presence of air, there are two main peaks: (i) a more intense peak at 2θ ∼ 27.5° (*d*_001_ = 0.324 ± 0.001 nm), which corresponds to the interplanar distance within the stack of carbon nitride layers and (ii) a less intense peak at 2θ ∼ 13.0°, which corresponds to (210) and (-210) planes with an interplanar distance of ∼0.680 ± 0.001 nm, reflecting the arrangement of heptazine blocks in carbon nitride layers^25^ (Figure 1a,b). In addition, the (310) and (310) reflection at 2θ ∼ 18° and several broad features at ∼10° and ∼44° are visible. The observed diffraction patterns are characteristic of bulk polymeric carbon nitride and confirm the formation of this phase in our conditions.^21,26^

Treatment of g-C_3_N_4_ in molten salts leads to a rearrangement of heptazine blocks within melon sheets of g-C_3_N_4_ and the formation of PHI, hereafter denoted as K-PHI (Figure 1a). The obtained diffraction pattern of K-PHI can be indexed in a trigonal unit cell as described earlier.^27^ In the 2θ region 24–32°, two characteristic peaks of K-PHI are present, 001 and 101, which are much sharper than the broad 001 peak of g-C_3_N_4_. At 2θ = 8.1°, a sharp 100 peak is observed, thus indicating an increase in crystalline order within each layer and in the layer stacking during salt melt treatment. Treatment of K-PHI in an acid solution or irradiation of the K-PHI dispersion in the presence of acid and platinum ions leads to a change of its structure (Figure 1a,b; H-PHI and H-PHI/Pt, respectively). In particular, the appearance of the additional peak at 2θ ∼ 11° and a shift of the 001 peak to 28.2° point toward a distortion of the trigonal symmetry and the formation of the H-PHI structure with considerable disorder as described by Schlomberg et al.^28^ It should be noted that the diffractogram of H-PHI/Pt is nearly identical to that of H-PHI. The absence of a peak at 2θ = 39.8°, which otherwise is assigned to the (111) plane of face-centered cubic Pt in the diffraction patterns of H-PHI/Pt, is due to the low platinum content in the sample.^29^

The TEM study confirmed the structural analysis results of g-C_3_N_4_, K-PHI, H-PHI, and H-PHI/Pt obtained from X-ray powder diffraction. As shown in Figure 3a,b, g-C_3_N_4_ consists of micrometer-sized particles, which show no long-range order. The K-PHI sample mainly consists of elongated particles with a length of about 100–200 nm and a width of about 10–20 nm (Figure 3c,d) that are assembled into large agglomerates. The morphology of these agglomerates suggests that K-PHI crystals are probably bound to each other through a joined carbon nitride layer on which they are seeded. Lattice fringes on high-resolution TEM (HRTEM) images of K-PHI particles (e.g., Figure 3d) reflect the ordering in the K-PHI layers. The averaged *d*_100_ spacing is found to be equal to 10.45(30) Å. Treatment of K-PHI in acid solution, both in the dark and under irradiation, does not lead to any significant changes in the morphology of the samples (Figure 3e–h). However, we observed a considerably high number of disordered crystallites and a decrease in the *d*_100_ spacing to 9.89(55) Å. The latter most probably reflects the distortion of the trigonal crystal lattice toward a less symmetrical one during ion exchange of K^+^ ions by H^+^/H_3_O^+^ ions. Platinum particles are randomly distributed in the sample and have an average size of 1.86 ± 0.51 nm.

**Figure 2:**
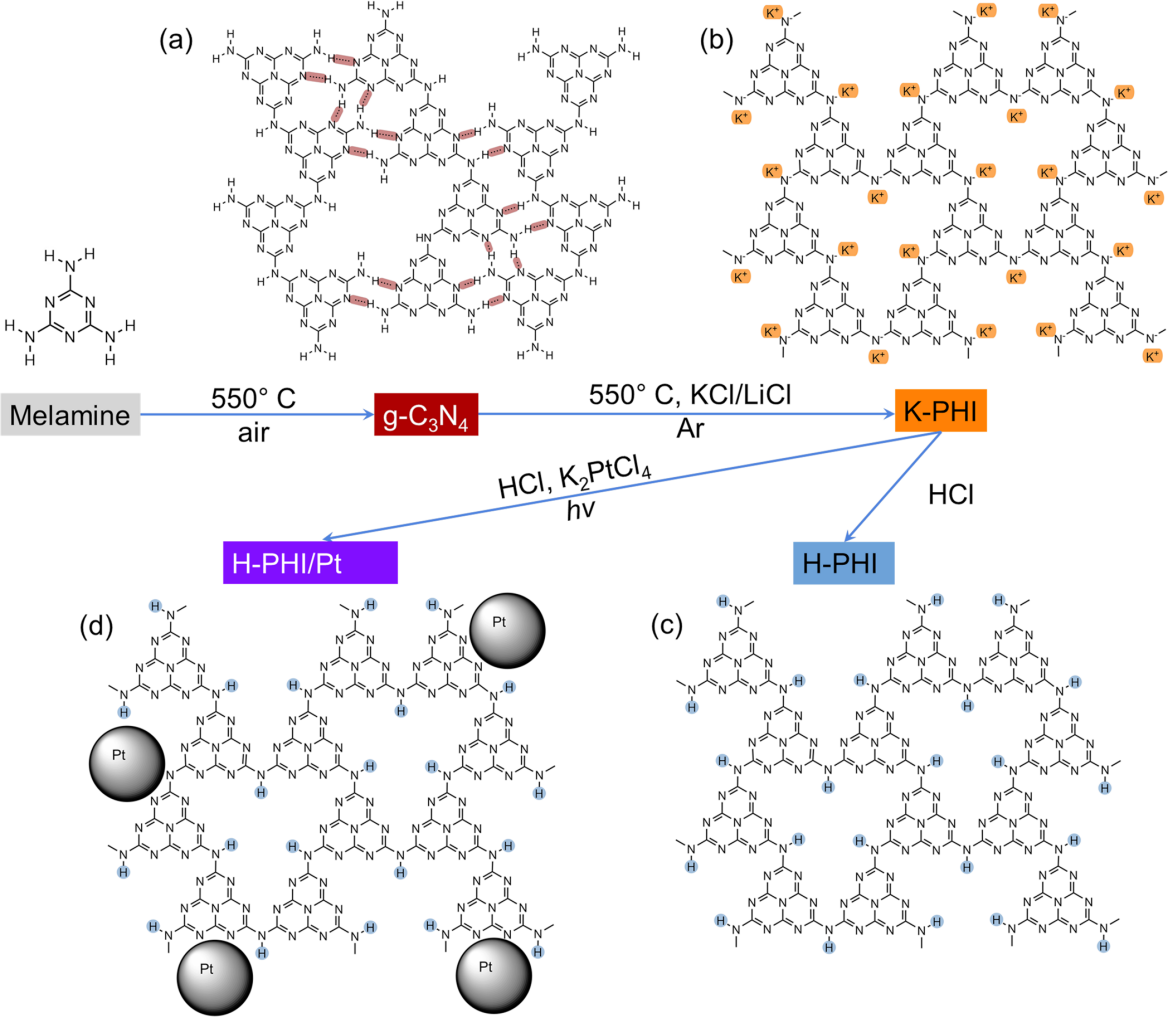
Schematic representation of the synthesis process and structure of the obtained photocatalysts. (a) g-C_3_N_4_, (b) K-PHI, (c) H-PHI, and (d) H-PHI/Pt.

**Figure 3:**
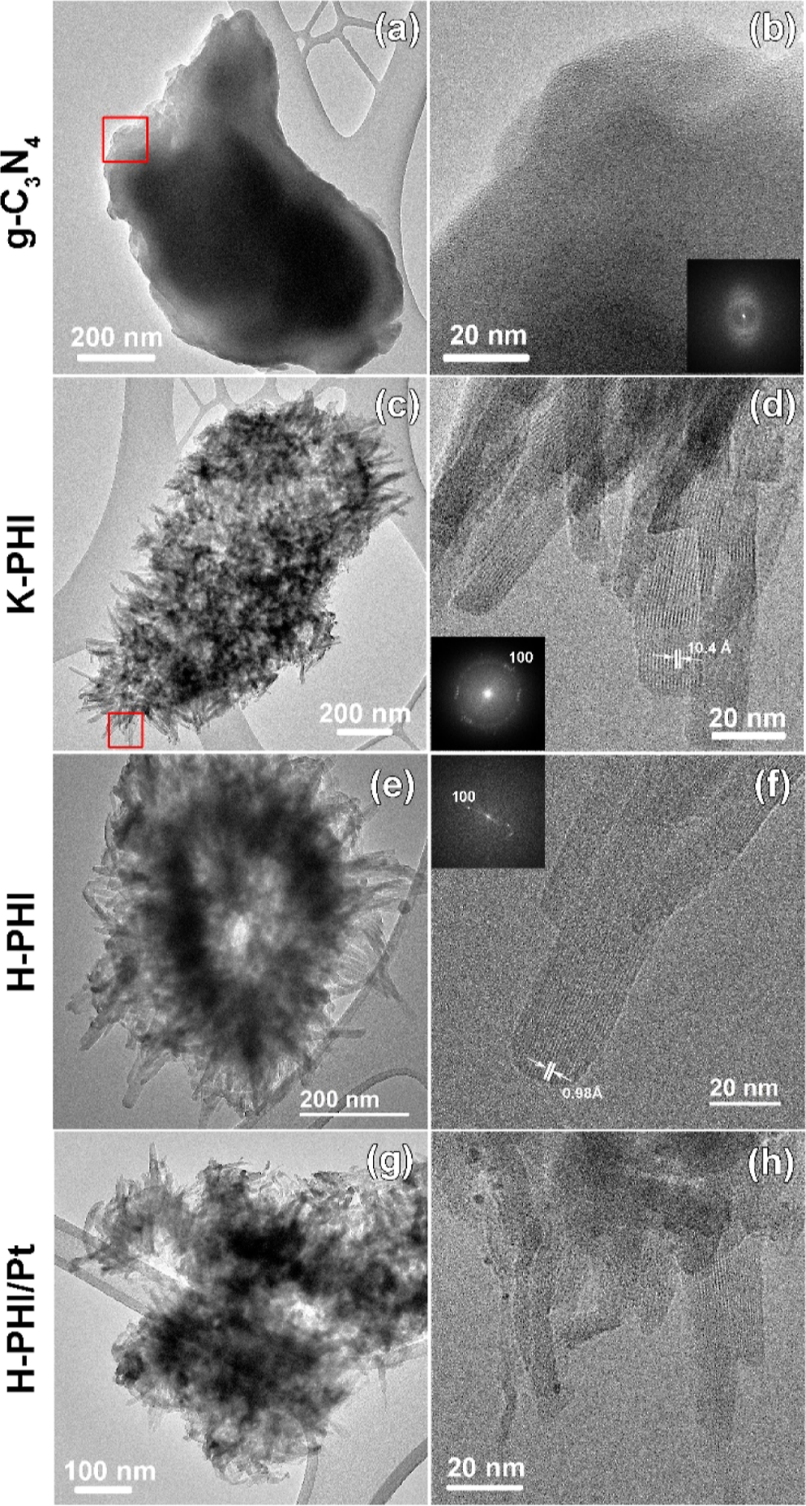
Overview and HRTEM images of (a,b) g-C_3_N_4_, (c,d) K-PHI, (e,f) H-PHI, and (g,h) H-PHI/Pt samples. Red squares mark areas of the overview TEM images that are enlarged on the corresponding HRTEM images. Insets show the fast Fourier transformations obtained from the corresponding HRTEM images.

In FT-IR spectra (Figure 1c,d), all the obtained materials show a peak at 808 cm^–1^, the so-called “fingerprint” of the heptazine unit,^30,31^ which indicates that all the studied samples are heptazine-based carbon nitrides. Also, all samples contain a set of peaks in the region of 1200–1700 cm^–1^, which are characteristic to the vibrations of the tri-*s*-triazine derivatives.^17,32^ In addition, all the samples contain a broad peak in the region of 3000–3500 cm^–1^, characteristic to terminal amino groups and hydroxyl groups.^17^ However, a detailed analysis of the FT-IR spectra shows a number of fundamental differences between the obtained samples. In particular, the IR spectrum of g-C_3_N_4_ contains a peak at 3100 cm^–1^, which, according to the literature, may indicate the presence of a significant number of hydrogen bonds between hydrogen in terminal amino groups and nitrogen in heptazine units.^33^ The presence of such hydrogen bonds is caused by the imperfection of the structure and is schematically shown in Figure 2a (highlighted with brown). Hydrogen bonds in the structure of carbon nitride can act as recombination centers of photogenerated charges and reduce the photocatalytic activity of such a material.^32^ Treatment of g-C_3_N_4_ in a molten salt, as well as further treatment in an acid solution, leads to a shift of the specified peak to ∼3367 cm^–1^, which may indicate formation of a more rigid structure—wavenumber scales with the force constant, ν ∼ *k*^1/2^,^33^ which is due to a more perfect packing of heptazine units in the structure of carbon nitride (Figure 2b–d). All of the obtained crystalline samples of carbon nitride contain a peak at 2182 cm^–1^. This peak is due to the formation of terminal cyano groups^33,34^ during additional thermal treatment in molten salts. The cyano groups are preserved in acidic medium and light irradiation. According to the literature, such groups can facilitate charge separation and accumulation,^34^ and therefore increase the photocatalytic activity of such materials. In addition, there are two peaks at 986 and 1115 cm^–1^ in the FT-IR spectrum of K-PHI, which can be attributed to symmetric and asymmetric vibrations of metal-NC_2_ groups, respectively (Figure 1c,d).^35^ The presence of these peaks in the spectrum of K-PHI confirms the entry of potassium ions into the carbon nitride structure during treatment in molten salts. Absence of these peaks in the FT-IR spectra of H-PHI and H-PHI/Pt confirms removal of K^+^ during the acid treatment. Attention is drawn to the almost complete disappearance of the peak at 1408 cm^–1^ upon the treatment of g-C_3_N_4_ in molten salts. This peak corresponds to the oscillation of N–H groups that link heptazine groups (bridging nitrogen).^36^ The disappearance of this peak in the IR spectrum of K-PHI may be associated with the replacement of hydrogen in the bridging N–H groups by potassium and is consistent with the proposed structure of K-PHI (Figure 2b). At the same time, after treatment in an acid solution, the specified peak reappears in the H-PHI and H-PHI/Pt samples, which confirms the assumption that potassium is removed from K-PHI in an acidic medium and replaced with a proton. Also, treatment in acid leads to an increase of the intensity of peaks in the region 1440–1615 cm^–1^ that are associated with the vibration of aromatic rings,^36^ which is due to the removal of cross-linking hydrogen bonds, or potassium, which can electrostatically interact with nitrogen in several layers. The data obtained by the XRD and FT-IR methods are in good agreement with the proposed structure and composition of the materials obtained in the work (Figure 2).

**Figure 4:**
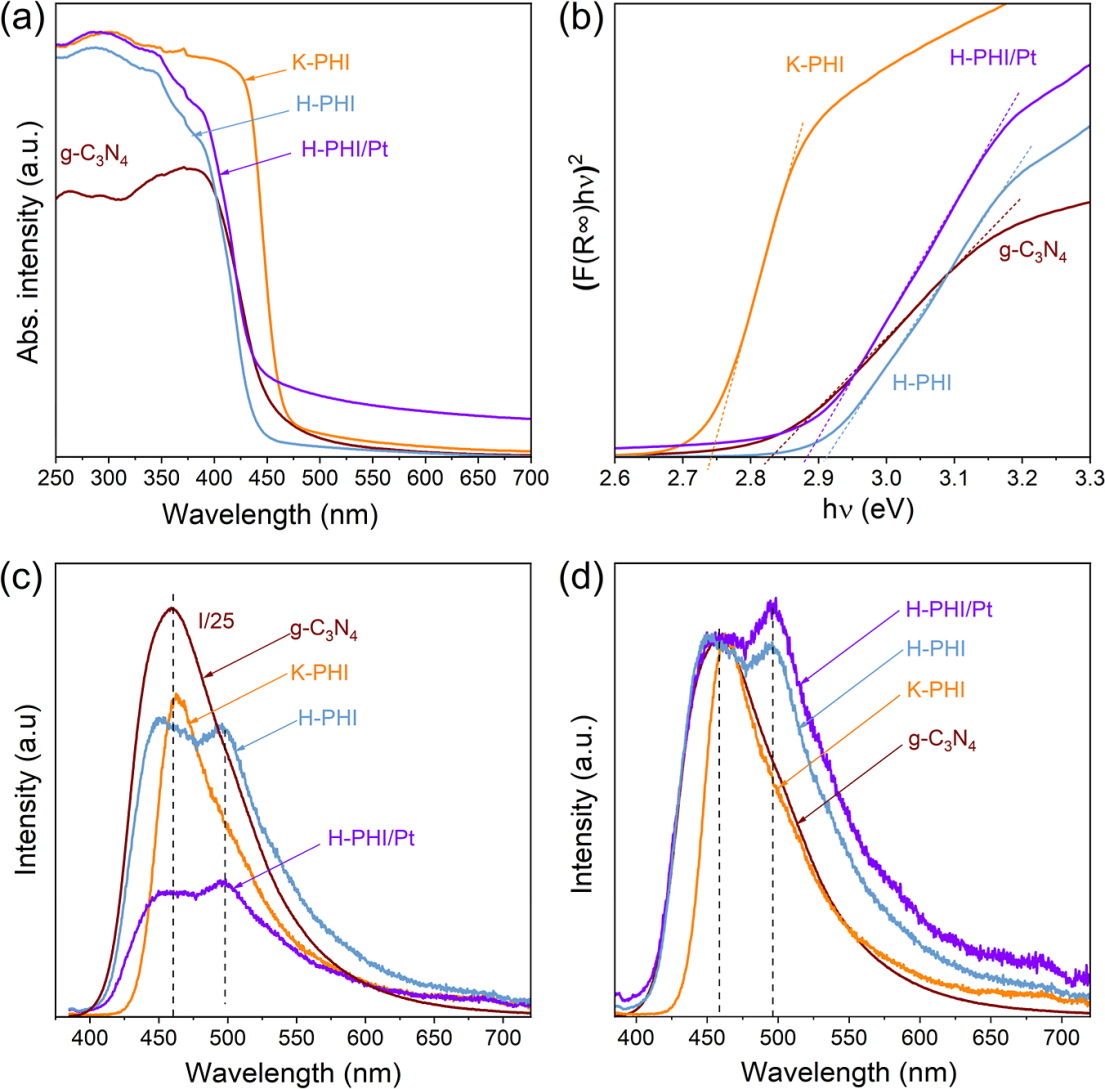
Absorption spectra (a), absorption spectra in the coordinates of the Tauc equations (b), photoluminescence spectra (c), and normalized luminescence spectra (d) of g-C_3_N_4_, K-PHI, H-PHI, and H-PHI/Pt.

In the UV–vis absorption spectrum of g-C_3_N_4_, there is an intense band with the absorption edge at about 450 nm (Figure 4a), which corresponds to the band gap (*E*_g_) of about 2.83 eV (Figure 4b) and is consistent with the literature.^10,26^ Treatment of the obtained g-C_3_N_4_ in the eutectic melt of KCl and LiCl salts at 550 °C for 4 h in a stream of argon leads to significant changes in the absorption spectrum (Figure 4a). Thus, the long-wavelength absorption edge of the treated material (K-PHI) undergoes a bathochromic shift of about 20 nm, which corresponds to a decrease of *E*_g_ to about 2.75 eV (Figure 4b). There is also a significant increase in the absorption intensity of the material treated in salts. These changes, according to the literature,^37^ indicate an increase in the length of the conjugation region in the carbon nitride and an increase in the orderliness of the structure of such a material.

Treatment of K-PHI by a diluted aqueous solution of hydrochloric acid leads to a hypsochromic shift of H-PHI absorption edge by about 30 nm relative to K-PHI, which corresponds to an increase of *E*_g_ to about 2.91 eV (Figure 4b). These changes are obviously due to the removal of incorporated potassium ions from the structure of carbon nitride during treatment in acid. At the same time, the absorption intensity of such a material remains practically unchanged compared to that of K-PHI and significantly exceeds the absorption of bulk g-C_3_N_4_. This is consistent with the literature and may be due to the preservation of high order in carbon nitride after treatment in acid. Irradiation of K-PHI in the presence of acid and platinum ions also leads to a hypsochromic shift of the edge of the absorption band (H-PHI/Pt) in Figure 4a, which corresponds to the increase of *E*_g_ to about 2.88 eV and confirms the same effect of the acid regardless of the conditions—either under irradiation or in the dark. At the same time, platinum ions are deposited on the surface of carbon nitride, followed by their reduction and formation of metal nanoparticles, which leads to the increase of absorption in the visible range of the spectrum.^29^

The thermal treatment of g-C_3_N_4_ in molten salts leads to a decrease in the luminescence intensity of the obtained material (K-PHI) by more than 30 times (Figure 4c), which may be associated with the removal of radiative recombination centers from the structure of carbon nitride. At the same time, the shape of the luminescence band and the position of its maximum (λ = 460 nm) remain practically unchanged. Such a significant decrease in the luminescence intensity can lead to an increase of the photocatalytic activity of the obtained material, since radiative recombination of the photogenerated charges is the reason that decreases the overall efficiency of the photocatalyst.^30^ In the H-PHI sample, the luminescence intensity is comparable to that of K-PHI, however, an additional luminescence maximum at about 497 nm is observed (Figure 4c,d). The energy of such a band is lower than the *E*_g_ value for this material, which indicates that this luminescence involves defects that result in the appearance of energy states within the band gap. However, given that the intensity of this band is low, we conclude that the number of defects associated with this band is relatively low. In the case of H-PHI/Pt, the formation of two maxima is also observed, but their intensity is lower compared to that of H-PHI. This additional decrease of the luminescence intensity is due to the transfer of the photogenerated electrons to Pt, which suppresses their radiative recombination with the holes.

The chemical compositions of the samples obtained in the work are shown in Table 1. These results confirm our assumptions about the structure and composition of the photocatalysts made above. In particular, the increase of the C/N ratio from 0.57 to 0.61 when moving from g-C_3_N_4_ to crystalline samples is due to the removal of the excess of terminal amino groups, which are considered defects (Figure 2a). It should be noted that during the treatment of g-C_3_N_4_ in the melt of KCl/LiCl salts sublimation and condensation of ammonium chloride were observed at the outlet of the tube furnace, which can confirm the removal of terminal amino groups during the calcination. Also, the K-PHI sample contains about 8.7 and 0.7 wt % of K and Li, respectively, which were incorporated into its structure during the thermal treatment in molten salts. Treatment with acid does not lead to a significant change of the C/N ratio, which suggests chemical stability of the K-PHI structure (Figure 2b, c). However, treatment of K-PHI with acid leads to an increase of the hydrogen content in the H-PHI and H-PHI/Pt samples from 2.7 to 3.3 wt %, which is due to the removal of K^+^ and Li^+^ from the K-PHI structure and their replacement by proton. In addition, the H-PHI/Pt sample, according to ICP data, contains 0.45 wt % of Pt, which is close to the theoretical value of 0.5 wt %, assuming quantitative reduction of Pt(+4) ions and their deposition at the H-PHI.

**Table 1:** Percentage of Elements in Carbon Nitride Samples and Their SSA^c^

sample	N^a^(wt %)	C^a^(wt %)	H^a^(wt %)	C/N	K^b^(wt %)	Li^b^ (wt %)	Pt^b^ (wt %)	SSA (m^2^ g^–1^)
g-C_3_N_4_	54.3	31.0	2.8	0.57				4.4
K-PHI	43.8	26.9	2.7	0.61	8.7	0.7		144.2
H-PHI	45.8	28.0	3.3	0.61				229.6
H-PHI/Pt	45.0	27.7	3.3	0.62	1.4	0.02	0.45	195.4

a Combustion analysis: C, H, and N.

b ICP–OES: K, Li, and Pt.

c The elemental composition data were obtained using combustion analysis and ICP spectroscopy.

It should be noted that the treatment of g-C_3_N_4_ in molten salts leads to a significant increase of the specific surface area (SSA) of the sample from 4.4 to 144.2 m^2^ g^–1^ (Table 1). This may be due to a significant change in the morphology of the sample. Treatment of K-PHI with acid leads to a further increase of the SSA to 229.6 and 195.4 m^2^ g^–1^ for H-PHI and H-PHI/Pt, respectively (Table 1), which may be due to partial delamination of the samples during their treatment in acid.

### 3.2 Life Cycle Assessment of Photocatalytic Acetal Production in Comparison to Benchmarks

We assess the environmental impacts of acetal production using life cycle assessment (LCA), an established methodology to quantify the environmental impacts of technologies and products.^38^ Thereby, we explore whether photocatalytic ethanol oxidation could reduce environmental impacts compared with industrial benchmarks. Typically, LCA would require detailed industrial data on the final photocatalysis process design, which is unavailable for the presented photocatalytic ethanol oxidation on the lab scale. To enable a prospective LCA,^39^ we thus scale up the photocatalysis process to a commercial scale using flowsheet simulations in Aspen Plus (version 11) based on the data obtained from the experiments (see Section 2.4) (Supporting Information) to calculate the life cycle inventory. In particular, we assume the conversion is currently achieved experimentally, which we regard as a conservative assumption. Thus, if the modeled photocatalysis has lower environmental impacts than the benchmarks, then the LCA results would encourage further development of acetal production from photocatalysis.

The photocatalytic acetal production could be benchmarked to alternative acetal-production technologies or other molecules with similar functionality that could be replaced by acetal. To our knowledge, no LCA study on acetal production is available in the literature, while several uses of acetal have been discussed in the literature. Here, we consider the use of acetal as a fuel additive, replacing methyl *tert*-butyl ether (MTBE),^40^ and as a solvent, replacing diethyl ether.^41^ To confirm these potential replacements, we assessed the thermodynamic properties (Supporting Information), which are similar on a molar basis. Thus, we assume that 1 mol of acetal replaces 1 mol of either MTBE or diethyl ether.

The LCA is based on the inventory data obtained from the process model, which includes ethanol feedstock and separation energy demands for producing 1 kg of acetal. Unreacted ethanol is recycled. Hydrogen and acetaldehyde are byproducts. To account for the same byproducts in the benchmarks, we apply system expansion by adding fossil hydrogen and acetaldehyde production to the MTBE and diethyl ether benchmarks. All processes are regarded as global in a cradle-to-grave assessment with the incineration of products at the end of life.

**Figure 5:**
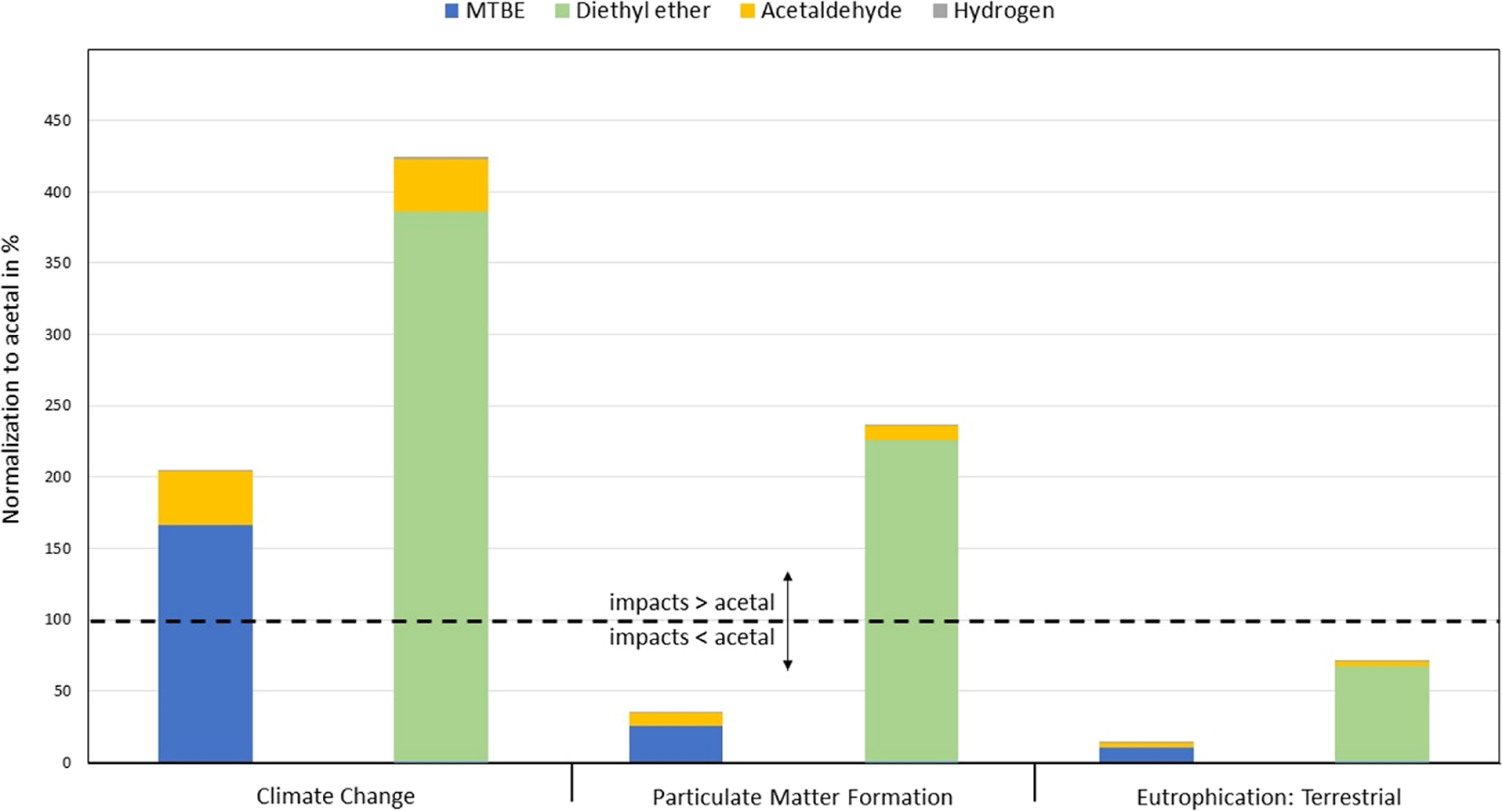
Environmental impacts of MTBE and diethyl ether compared to acetal in three exemplary impact categories where (i) MTBE and diethyl ether have higher impacts than acetal in climate change, (ii) only diethyl ether has higher impacts than acetal in particulate matter formation, and (iii) MTBE and diethyl ether have lower impacts than acetal in terrestrial eutrophication. Diethyl ether impacts are higher than MTBE impacts in all categories. Results for all impact categories are displayed in the Supporting Information. Benchmark impacts are above 100% when lab-scale acetal production from photolysis results in a reduction in environmental impact reduction. Benchmark impacts below 100% indicate that acetal production from photolysis has no potential to reduce environmental impacts compared to the benchmark.

The investigated photocatalysis process for acetal production can potentially reduce greenhouse gas (GHG) emissions by half, from 0.41 to 0.2 kg CO_2_-eq per mole when replacing MTBE, and even down to more than one-fourth from 0.85 kg CO_2_-eq per mole when replacing diethyl ether (Figure 5). The GHG emissions of MTBE production are mainly dominated by the feedstock production, i.e., butadiene and sulfuric acid, while the GHG emissions of the diethyl ether production result mainly from the feedstock production of sodium hydroxide and methanol. In contrast, the biobased ethanol feedstock of the photocatalysis process emits much lower GHGs, reducing the potential GHG emissions compared to the investigated benchmarks.

Overall, acetal production reduces environmental impacts in 10 of 16 impact categories compared with diethyl ether (see Supporting Information for details). In contrast, MTBE production results in lower environmental impacts than acetal production, except for global warming impacts and nonrenewable energy resources.

Thus, at the current stage, investigating acetal as a diethyl ether replacement seems most promising from an environmental perspective. Still, the MTBE replacement seems promising if the focus lies on GHG emissions and fossil resource reduction. However, researchers should monitor shifts in burden to other impact categories, especially marine and terrestrial eutrophication, human toxicity, land use, and water use. Since early stage LCA is intrinsically characterized by high uncertainty,^42^ the flowsheet simulations should be continuously updated with more detailed process data, such as reaction yield, selectivity, catalyst, and energy demands. Based on this information, the LCA should be revisited to confirm the environmental benefits of photocatalytic acetal and hydrogen production.

In summary, the LCA results for acetal production are encouraging, taking into account that lab-scale photocatalysis can still be optimized to reduce environmental impacts at the industrial scale.

### 3.3 Photocatalytic Activity

**Figure 6:**
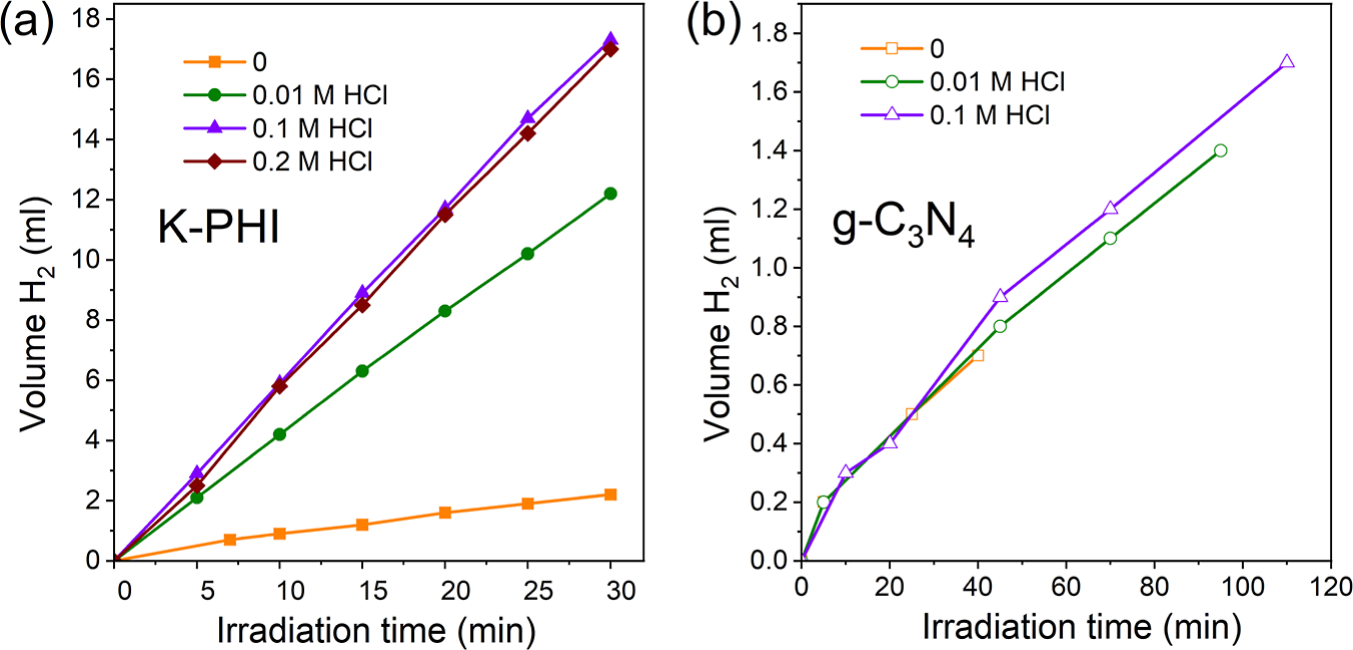
Kinetic curves of photocatalytic hydrogen formation depending on the concentration of hydrochloric acid in the system in the presence of K-PHI (a) or g-C_3_N_4_ (b). Conditions: photocatalyst (10 mg), K_2_PtCl_4_ (0.25 μmol), ethanol/water (5 mL, 96:4 wt %), λ = 410 nm (85 mW cm^–2^), and Ar atmosphere.

Irradiation of a deaerated ethanol suspension containing K-PHI and K_2_PtCl_4_ with visible light (λ = 410 nm) leads to the formation of hydrogen gas in the system (see Figure 6a, curve with square dots, and Table 2). The rate of hydrogen evolution in this case reaches about 18.2 mmol g^–1^ h^–1^ (Table 2). This value is high and under comparable conditions and irradiation wavelength ≥410 nm exceed performance of many photocatalytic systems applied to the conversion of alcohols (see Table S5(43) and review^18^).

**Table 2:** Rate of Photocatalytic Hydrogen Formation Depending on the Concentration of Hydrochloric Acid in the System in the Presence of K-PHI or g-C_3_N_4_

photocatalyst	[HCl], (mol L^–1^)	H_2_ evolution efficiency, (mmol g^–1^ h^–1^)
K-PHI	0	18.2
	0.01	100.7
	0.1	142.9
	0.2	140.4
g-C_3_N_4_	0	3.0
	0.01	3.0
	0.1	3.0

Taking into account that acid can significantly affect the efficiency of photocatalytic systems based on K-PHI, as well as to increase their stability,^21,44^ we studied this effect. Indeed, from the results shown in Figure 6a and Table 2, the addition of 0.01 mol L^–1^ acid leads to a significant increase in the rate of hydrogen production, which in this case reaches a value of about 100.7 mmol g^–1^ h^–1^. A further increase of the acid concentration in the reaction mixture leads to an increase of the rate of hydrogen production to about 142.9 mmol g^–1^ h^–1^, and practically does not change with a further increase of the acid concentration to 0.2 M (see Figure 6a, curve with diamond dots, and Table 2). The obtained value of the quantum yield of photocatalytic production of hydrogen is one of the highest known in the literature when using light in the visible range (Table S5).^18,43^ At the same time, the addition of acid to the system containing g-C_3_N_4_ does not lead to a change in the rate of hydrogen production (Figure 6b and Table 2) and is due to the high chemical stability of this material to dilute acid solutions.^30^

Such a significant increase in photocatalytic activity in the reaction of hydrogen evolution during the transition from g-C_3_N_4_ to K-PHI and then to H-PHI, 1.5, 18, and 143 mmol g^–1^ h^–1^, respectively, can be caused by a number of factors. In particular, by removing defects from the structure of the photocatalyst—terminal NH_2_-groups by calcining g-C_3_N_4_ in salts melt, they can act as recombination centers of photogenerated charges. Also, the activity is significantly affected by the decrease in the size of the particles and the increase in their crystallinity, which facilitates the transfer of photogenerated charges to the surface where they can participate in redox processes. An increase in the SSA of the photocatalyst, which improves the adsorption of substrates on the surface, reduces the negative impact of diffusion processes, and increases the specific content of surface active centers in activated photocatalysts, etc.

**Figure 7:**
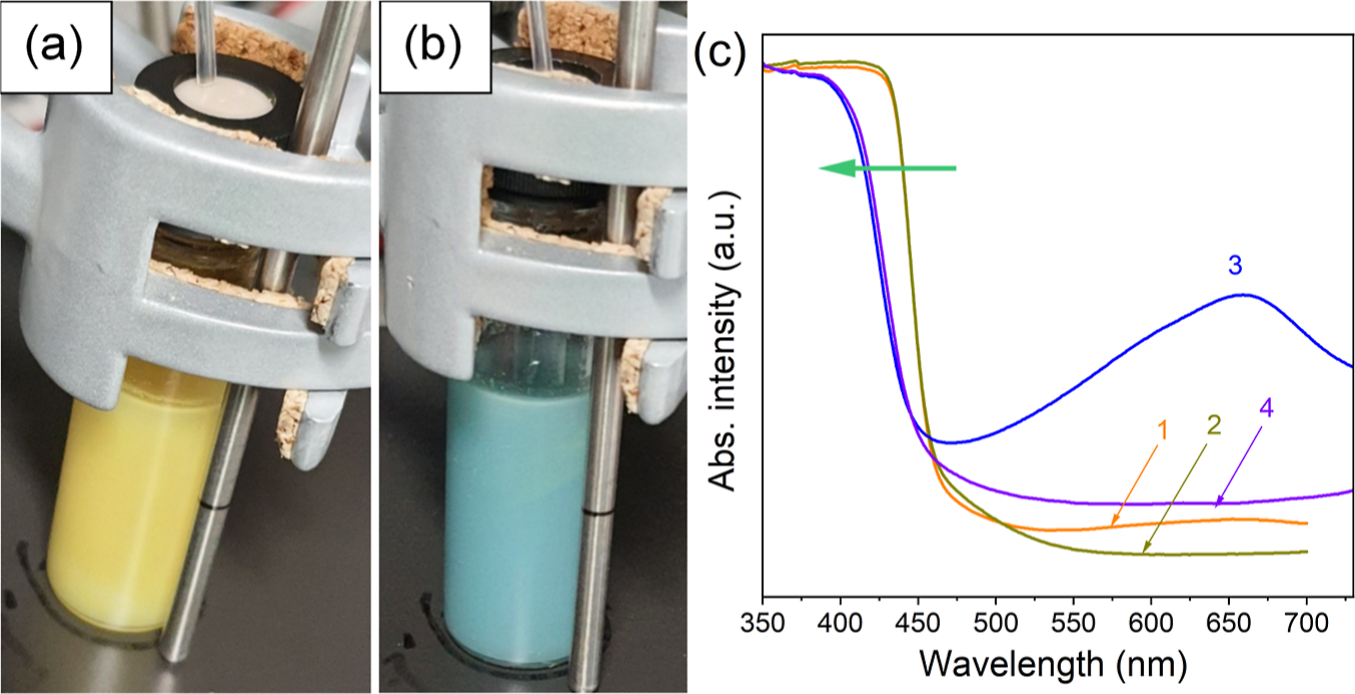
Photographs of vials containing irradiated for 30 min deaerated suspension of K-PHI without acid (a) and after injection of 0.01 M HCl into the solution (b). (c) Absorption spectra of deaerated suspension of K-PHI without acid before irradiation (curve 1), after irradiation for 30 min (2), after injection of 0.01 M HCl into the irradiated suspension (3), and after exposure to air (4). Conditions: K-PHI (10 mg), K_2_PtCl_4_ (0.25 μmol), ethanol/water (5 mL, 96:4 wt %), λ = 410 nm (85 mW cm^–2^), and Ar atmosphere.

We found that addition of acid to an ethanol suspension containing K-PHI and platinum ions in the dark, which prior was irradiated for 30 min, leads to a rapid change of carbon nitride color from bright yellow to blue (see Figure 7a,b).

Such a color change is manifested in the absorption spectrum of the suspension as a broad band in the entire visible range with a maximum at about 658 nm (Figure 7c, curve 3). The indicated color change and the appearance of the corresponding absorption band in the studied system may be associated with the accumulation of “free” electrons in the structure of carbon nitride or its photocharging.^45,46^ The introduction of air into the system leads to a rapid discoloration of the suspension and the disappearance of the corresponding absorption band (see Figure 7c, curve 4), which confirms our assumption that this band is due to the accumulation of strongly reductive electrons in the system upon the addition of acid since such electrons can easily react with oxygen from the air and be removed from the semiconductor. The fact that the appearance of the indicated band does not require additional irradiation after the addition of acid is noteworthy. This band is formed in the dark after adding acid to the irradiated suspension. Therefore, the mode of color induction is very different compared to that reported in many publications earlier.^46^ This may indicate that irradiation of K-PHI without acid leads to the formation of the long-lived locally excited states in the structure of the photocatalyst, and the acid promotes only the separation and transport of such charges to the surface of the photocatalyst. When acid is added to the irradiated ethanol solution containing K-PHI and K_2_PtCl_4_, in addition to the appearance of a band with a maximum at 658 nm, a hypsochromic shift of the long-wave absorption band of the semiconductor is observed (indicated by the arrow in Figure 7c). This shift in the absorption band persists after air is introduced into the system (Figure 7c, curve 4) and may indicate the removal of potassium from the structure of the photocatalyst at the same time (Figure 4a).

Based on the obtained data and earlier reports,^47^ we assume that K-PHI has low electronic conductivity,^48^ but because it contains oppositely charged centers N^–^–K^+^ (see Figure 2b), and according to reference,^49^ it behaves as an ion conductor. In this case, the photogenerated charges are actually blocked in the volume of the photocatalyst, and only surface groups, from which transport (or tunneling) to the substrates is possible, take part in the photocatalytic reaction. When acid is added to such irradiated K-PHI, potassium is replaced by a proton, which leads to a change of the ionic structure of the semiconductor to a covalent one, which increases its electronic conductivity^48^ and leads to the rapid separation and transport of photogenerated charges to the surface and their participation in the redox processes. When acid is added to a system containing irradiated g-C_3_N_4_, the photocatalyst does not turn blue, indicating that electrons do not accumulate on its surface or within its bulk during irradiation.

The obtained results are in good agreement with the data of the EPR study at room temperature. As can be seen from Figure S7a, all materials are EPR-silent prior to light irradiation. Irradiation of the samples at 410 nm for 5 min leads to the appearance of an intense EPR signal in the spectra of all crystalline carbon nitride samples (Figure S7b). This signal indicates the accumulation of unpaired electrons in the samples upon irradiation^50−52^ and is caused by the rapid interaction of photogenerated holes with ethanol. At the same time, as shown in Figure S7b, the highest signal intensity is observed for the K-PHI sample, where the photogenerated electrons remain trapped in the photocatalyst lattice due to its low conductivity, as mentioned earlier. The EPR signal intensity of H-PHI and H-PHI/Pt is significantly lower than that of K-PHI, which may be due to the easier transfer of electrons to the photocatalyst surface, where they can participate in redox processes with substrates adsorbed on the semiconductor surface. In the case of g-C_3_N_4_, the EPR signal is absent even after irradiation, which may be due to the rapid recombination of photogenerated charges in this material and is consistent with the luminescence study data, according to which the photogenerated charges in g-C_3_N_4_ efficiently recombine (Figure 4c).

**Figure 8:**
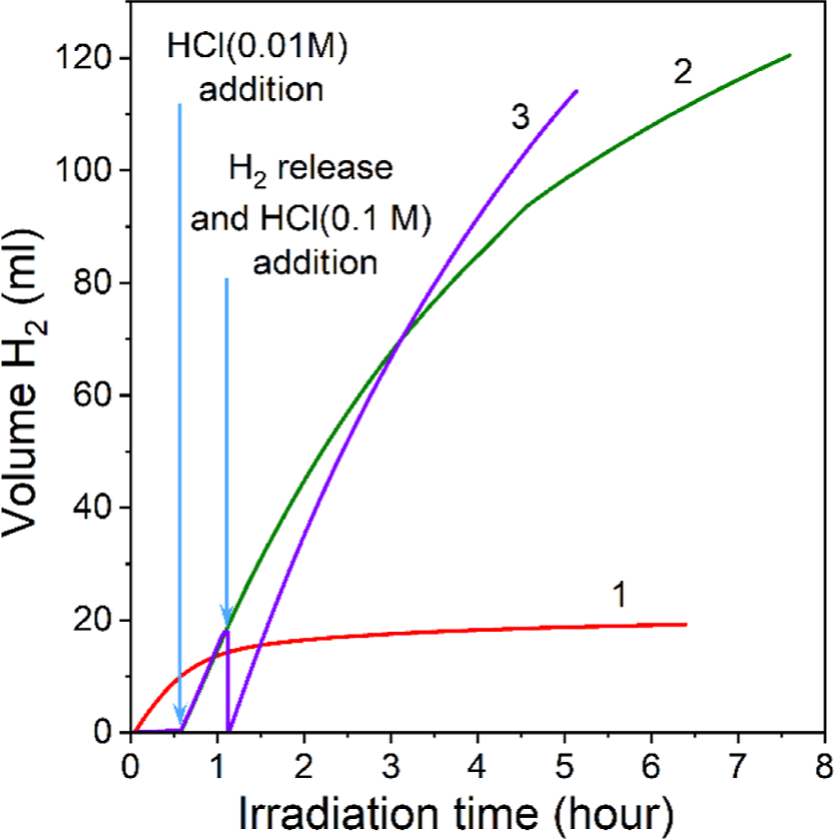
Kinetic curves of obtaining molecular hydrogen during the irradiation of a K-PHI suspension. Acid (HCl (0.01 M)) was added into the reactor before light irradiation (curve 1). Reaction mixture was irradiated with light for 30 min followed by addition of HCl (0.01 M) (curve 2). Reaction mixture was irradiated for 30 min followed by addition of HCl (0.01 M), irradiation for 30 min, and addition of HCl (0.1 M) (curve 3). Conditions: K-PHI (10 mg), K_2_PtCl_4_ (0.25 μmol), ethanol/water (5 mL, 96:4 wt %), λ = 410 nm (85 mW cm^–2^), and Ar atmosphere.

We found that the activity of the PHI-based photocatalytic system and its stability in the reaction of photocatalytic production of hydrogen from ethanol solutions are significantly affected by the conditions of its activation by acid. In particular, when we mixed K-PHI, platinum ions and acid with a water–ethanol solution and irradiated the mixture, we observed rapid evolution of hydrogen within first 0.5–1 h (Figure 8, curve 1). However, the reaction rate of hydrogen evolution gradually decreased, and after irradiation of such a system for about 1–1.5 h, the formation of hydrogen practically stopped. We observed a similar behavior when using H-PHI instead of K-PHI as a photocatalyst. Therefore, the addition of acid into the reactor before irradiating with light has a negative effect on the performance of the photocatalysts.

**Figure 9:**
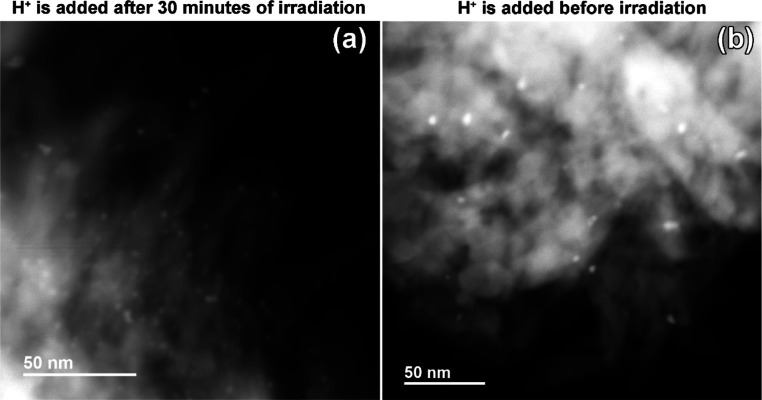
Annular-dark field STEM images of the H-PHI/Pt obtained when (a) acid was added to the suspension of K-PHI and platinum ions, preirradiated for 30 min, and when (b) acid was added into the system before irradiation. Pt particles appear as bright dots on the PHI matrix as the *Z*-contrast dominates the ADF-STEM images.

When we first irradiated a mixture of K-PHI and platinum ions for 30 min and after that added HCl (0.01 M), we detected a sharp increase in the rate of hydrogen formation, while the activity decreased very slowly (Figure 8, curve 2). Accumulation of hydrogen with high efficiency continued even after many hours of irradiation. Increasing the concentration of acid to 0.1 M in the reaction mixture, which was added to the preirradiated K-PHI, led not only to an increase of the activity of the system but also improved its stability (Figure 8, curve 3). We assume that the reason for such behavior can be explained by the difference in the accumulation of photogenerated charges in the volume of the photocatalyst. When the acid is added to the irradiated mixture of K-PHI and platinum ions, “locked charges” quickly migrate to the surface of the photocatalyst and form an increased number of photocatalytic-deposition centers for platinum nanoparticles. The platinum nanoparticles are then in situ deposited on these centers upon irradiation. An increased number of platinum deposition centers should lead to a reduction of the metal particles’ size and a more uniform distribution of them over the surface of the photocatalyst. When H-PHI is used as a photocatalyst directly, irradiation leads to the gradual release of photogenerated charges to the surface of the photocatalyst and the formation of platinum clusters there. Subsequent deposition of additional amounts of platinum will occur on the surface of already existing clusters. This should lead to an increase in the size of the platinum particles. Indeed, based on the analysis of the ADF-STEM images, the average size of the platinum particles that were deposited at H-PHI is twice larger (4.20 ± 0.44 nm) compared to the average size of the Pt particles deposited on the surface of K-PHI during irradiation and the subsequent addition of the acid (1.86 ± 0.51 nm); see Figure 9. Taking into account these results, in all subsequent experiments, acid was added to the system only after preliminary irradiation.

**Figure 10:**
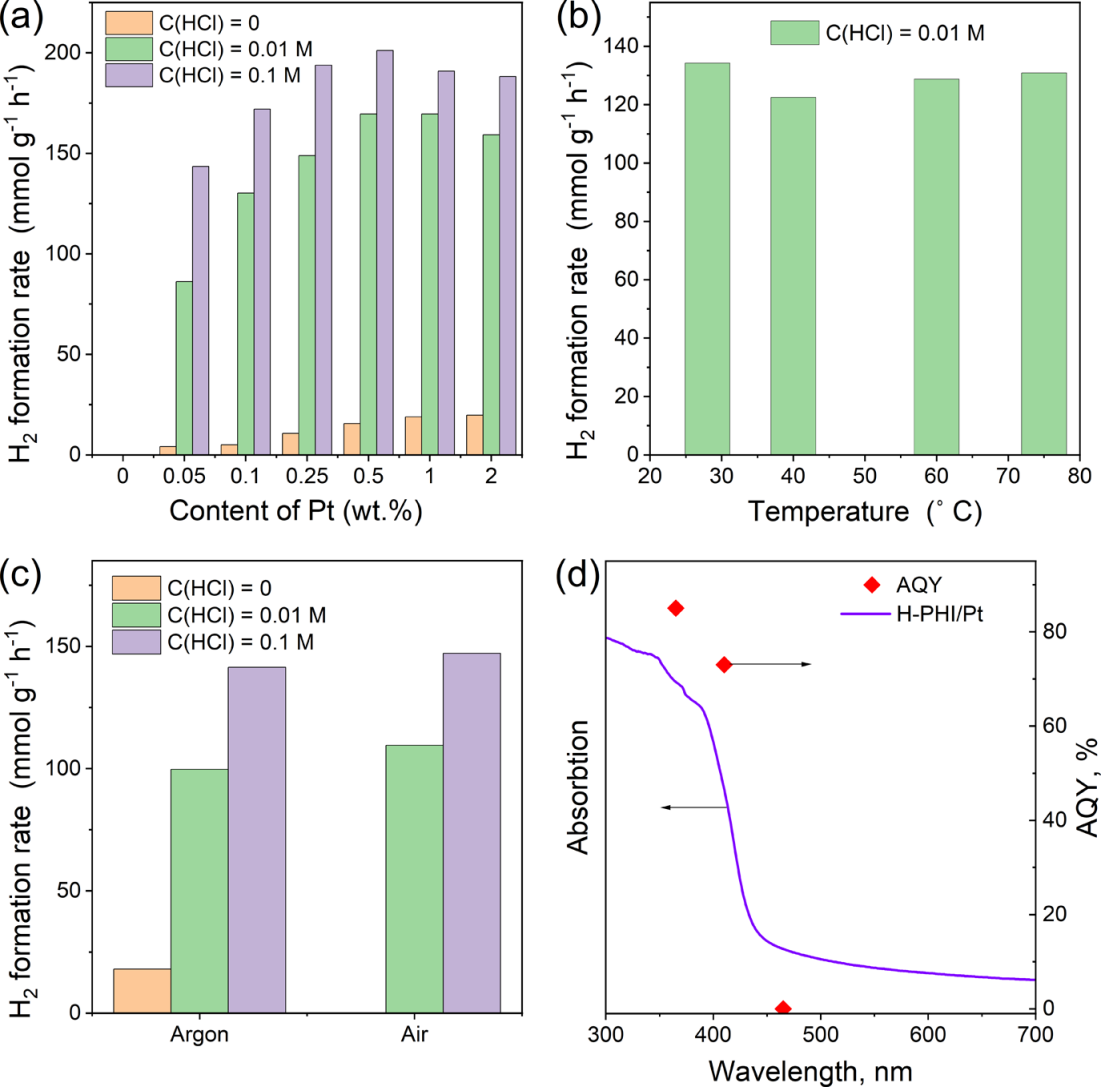
Dependence of the rate of hydrogen formation mediated by K-PHI on the content of platinum in the system in the absence of HCl and different final concentrations of HCl in the reaction mixture (a). Dependence of the H_2_ yield rate on the temperature of the reaction mixture (b). Dependence of the H_2_ yield rate on the presence or absence of air in the reactor (c). Wavelength dependence of AQY of H_2_ (diamonds) and UV–vis absorption spectrum of H-PHI/Pt (0.5 wt %) (curve) (d). Conditions: photocatalyst (10 mg), K_2_PtCl_4_ (0.25 μmol), ethanol/water (5 mL, 96:4 wt %), λ = 410 nm (85 mW cm^–2^), *C*(HCl) = 0.1 M, and Ar atmosphere.

To determine the optimal operating conditions of the investigated photocatalytic system, we studied the influence of a number of factors on the effectiveness of the most active photocatalysts. In particular, we investigated the dependence of the photocatalytic activity of K-PHI in the process of hydrogen formation from ethanol on the content of the cocatalyst in the system. Figure 10a shows that during light irradiation, H_2_ is not formed in the absence of platinum in the system. With the introduction of even 0.05 wt % Pt in relation to the mass of the photocatalyst, hydrogen begins to accumulate during the irradiation even in the absence of acid. At the same time, an increase in the content of platinum in the range of 0.05–2 wt % leads to a gradual increase in the rate of hydrogen formation (Figure 10a).

The addition of acid leads to a significant increase in the activity of the photocatalytic system (Figure 10a). The dependence of the rate of hydrogen formation on Pt content in the system in the studied range has the form of a curve with a maximum at 0.5 wt %, and it is independent of the acid content in the range of final concentrations 0.01–0.1 M. The decrease of H_2_ yield rate with the increase of the platinum content above 0.5 wt % may be due to a decrease in the activity of platinum nanoparticles with an increase of their size, a deterioration of the contact between the semiconductor and the photocatalyst in this case, as well as blocking the access of light to the photocatalyst with a high content of platinum since the platinum particles absorb light in the entire visible range. Even at the lowest Pt content in the system (0.05 wt %), in the presence of 0.1 M HCl, the rate of hydrogen evolution is high, and is only about 25% lower than at the optimal content of platinum. That is, a decrease in the content of platinum in the system by an order of magnitude from 0.5 to 0.05 wt % leads to only a slight decrease of the activity. At the same time, such a platinum content of 0.05 wt % is 20 times lower than the usually used in photocatalytic synthesis of 1,1-diethoxyethane from ethanol and up to 50 times lower in the photocatalytic conversion of alcohols (Table S5).^18,43^

We found that the activity of the studied photocatalytic system in the production of molecular hydrogen practically does not depend on the temperature in the range 28–75 °C (Figure 10b). The specified result may be due to rapid desorption of reaction products from the surface of the photocatalyst and negligible (∼0 kJ mol^–1^) thermal activation energy (*E*_a_) of this process.^53^ It must be lower than the apparent activation energy derived from pseudo Arrhenius plots of several dehydrogenation reactions. For example, in synthesis of benzylcyanide and H_2_ from benzene and acetonitrile over Pd/TiO_2_*E*_a_ = 43 kJ mol^–1^,^54^ in synthesis of H_2_ and succinonitrile from acetonitrile over Pd/TiO_2_*E*_a_ = 5.5 kJ mol^–1^,^54^ in synthesis of phenol and H_2_ from benzene and water *E*_a_ = 41 kJ mol^–1^,^55^ and in coupling of benzene with diethyl ether *E*_a_ = 35.4 kJ mol^–1^.^56^

Since for the potential practical application of photocatalytic hydrogen production it will be technologically costly to deaerate the reaction mixture, we investigated the efficiency of such a system in the presence of air. The results are shown in Figure 10c. In particular, we found that irradiation of the reaction mixture in the closed reactor, which was not degassed, without the addition of acid does not lead to the formation of hydrogen. Oxygen molecules in this case effectively capture photogenerated electrons^57^ and suppress their participation in the reaction of hydrogen evolution. At the same time, the introduction of 0.01 M acid into the system leads to the formation of hydrogen with high efficiency, which practically coincides with the efficiency of the system under anaerobic conditions. This result is related to the high efficiency of the system, which leads to the rapid removal of oxygen from the photocatalyst suspension and the blocking of its further access to the surface of the photocatalyst by hydrogen molecules formed there. Indeed, taking into account the solubility of oxygen in ethanol, there are 9.7 × 10^–6^ mol (O_2_) in 5 mL of the liquid phase.^58^ The amount of oxygen in 3 mL of the gas head space of the reactor is about 27 × 10^–6^ mol (O_2_). The number of photons that reach the reactor surface is 1.1 × 10^–6^ mol per second. Therefore, it takes only about 33 s to remove all oxygen from the liquid and gas phase parts of the reactor. When the acid content in the system is increased (Figure 10c), the yield rate of hydrogen increases, and it is close to that observed under anaerobic conditions with the same acid content. The obtained result is important and proves the possibility of effective operation of the studied system in a closed reactor without the need for degassing of the reaction mixture and additional deposition on the surface of the photocatalyst of the components that block the access of air,^59^ which in the future can significantly simplify the practical implementation of such photocatalytic systems to generate hydrogen.

**Figure 11:**
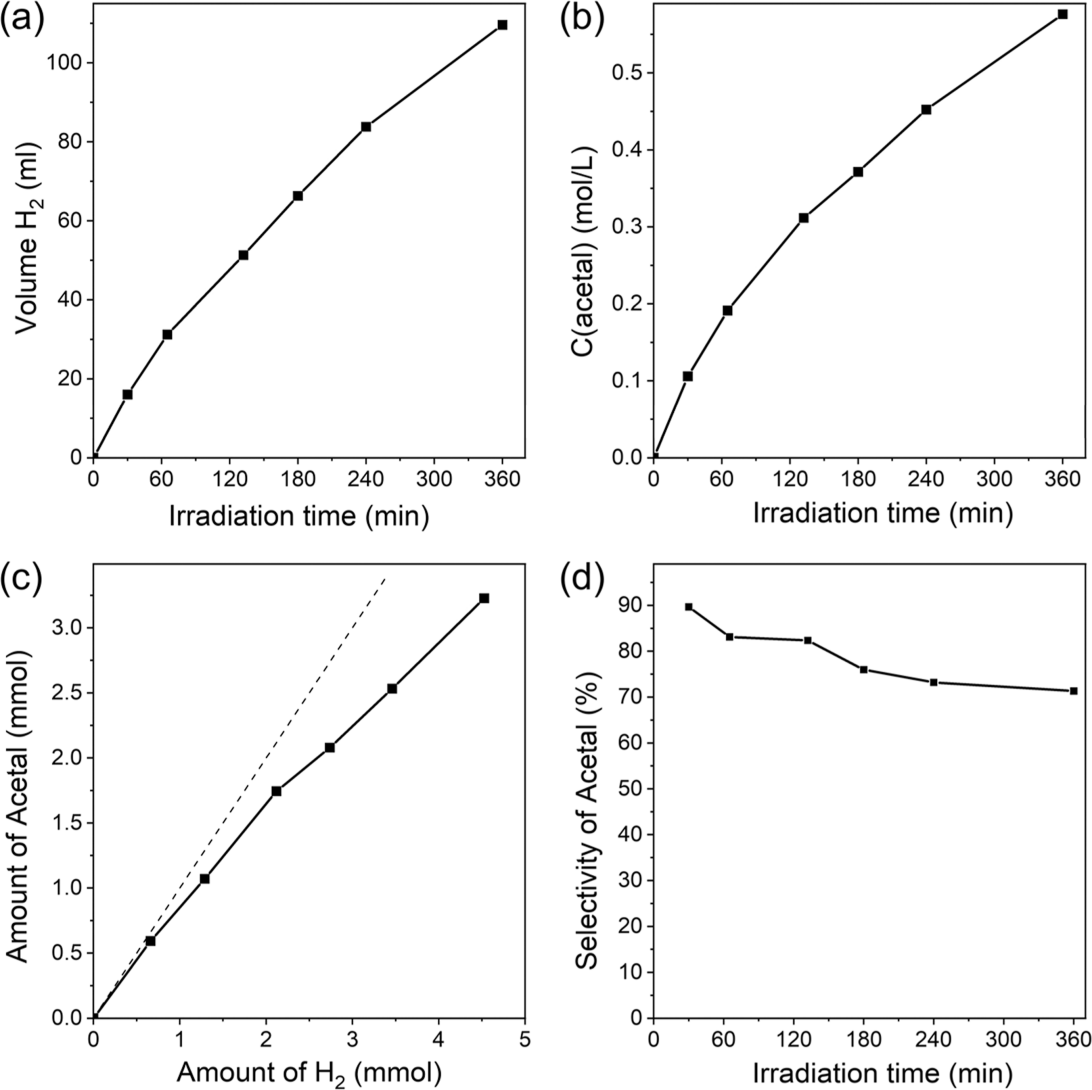
Kinetic curves of simultaneous photocatalytic hydrogen (a) and acetal (b) formation. Ratio between the amount of hydrogen and acetal formed during the photoreaction (c). Selectivity of acetal formation in solution (d). Conditions: photocatalyst (10 mg), K_2_PtCl_4_ (0.25 μmol), ethanol/water (5 mL, 96:4 wt %), λ = 410 nm (85 mW cm^–2^), *C*(HCl) = 0.1 M, and Ar atmosphere.

The activity of the studied system in the hydrogen production reaction significantly depends on the wavelength of the incident light and correlates with the absorption spectrum (Figure 11d). AQY of hydrogen production from water–ethanol solutions under visible light irradiation (λ = 410 nm) is 73%, which is one of the highest values in the literature (Table S5).^43^ The use of UV light leads to an even higher activity AQY (λ = 365 nm) of hydrogen production of 85% (Figure 10d). At the same time, increasing the irradiation wavelength to 465 nm leads to an almost complete absence of system activity (Figure 10d), which is due to the absence of light absorption by the photocatalyst in this region of the spectrum.

### 3.4 Photocatalytic Oxidation of Ethanol

The photocatalytic decomposition of water into hydrogen and oxygen is a thermodynamically challenging reaction, and the oxygen that is the product of the water oxidation half reaction not only has low cost but also forms an explosive mixture with hydrogen and requires a complex separation procedure.^13^ Considering this aspect, we carried out H_2_ generation from water–ethanol mixtures. The presence of ethanol in the system can not only significantly increase the efficiency of the photocatalytic process due to the lower oxidation potential of ethanol compared to water,^60,61^ but it also excludes the formation of oxygen under these conditions. Also, the oxidation of ethanol can purposefully lead to the formation of value-added products. That is, the use of ethanol additives can not only increase the efficiency and safety of the process of photocatalytic hydrogen production but also form products with added value, which can lead to greater economic efficiency of such a photocatalytic system as a whole.

Indeed, molecular hydrogen is formed in the gas phase (Figure 11a), while 1,1-diethoxyethane (acetal) is generated in the liquid phase, when a water–ethanol mixture containing H-PHI/Pt in the presence of HCl is irradiated with visible light (see Figures 11b and S8). In addition, it is possible to record a decrease in the ethanol content and the formation of acetaldehyde as the intermediate in the solution (Figure S8). The chemical structure of the ethanol oxidation product, acetal, was confirmed by recording the chromato-mass spectrum of the indicated peak (Figure S8, inset), as well as by recording ^1^H NMR (Figure S9) and ^13^C NMR spectra (Figure S10) of the reaction mixture after irradiation. Considering the acetal content in the solution and the selectivity of its formation after 360 min of irradiation, the conversion of ethanol in this case is 16.7 wt %.

According to the literature, acetal is one of the most important products of ethanol oxidation and has added value and wide practical application, in particular, in the chemical, pharmaceutical, and perfume industries. It can also be applied as an additive to diesel fuel, etc.^24^ It should be noted that the separation of acetal from ethanol is challenging due to the ability of these components to form an azeotropic mixture. However, given the potential of both chemicals to serve as fuel additives, their mixtures could be used for this purpose directly without the need for separation.

To evaluate the excited state dynamic of photocatalysts, we conducted fs-TA and transient photoluminescence decay measurements were conducted. The measurements were performed in acetonitrile (MeCN)—a polar solvent that affords a stable dispersion and typically does not scavenge the photogenerated electrons and holes. In addition, measurements were performed in ethanol—a solvent in which we conducted all of the photocatalytic experiments and which acts as the hole scavenger. Unless otherwise specified, the measurements were conducted in the presence of HCl (0.1 M) to mimic the conditions of the photocatalytic experiments. In case of K-PHI, H-PHI, and H-PHI/Pt suspensions in MeCN, in fs-TA spectra, the excited-state absorption (ESA) signal is observed at 600–800 nm and the stimulated emission (SE) at 500–600 nm, while for g-C_3_N_4_, the ESA is located at 500–650 nm and SE is at 700–800 nm (Figures S12–S20). Differently, in ethanol suspension, only the negative SE band at 500–650 nm appears. The early stage dynamic of the sample is analyzed by the single-wavelength kinetic fitting of the ESA for the suspension in MeCN and the SE for the suspension in ethanol. For the situation in MeCN, single-exponential fitting results in one lifetime, and the τ_1_ are 1.41, 1.31, 1.05, and 0.41 ps for g-C_3_N_4_, K-PHI, H-PHI, and H-PHI/Pt, respectively (Table S6). In ethanol that acts as a hole scavenger, the lifetime of the first excited-state species is longer than in MeCN, 2.56 4.65, 4.24, and 4.15 ps. These results suggest that recombination of the photogenerated hole and electron^62^ is suppressed in carbon nitrides featuring ionic structures, K-PHI, H-PHI, and H-PHI/Pt. Lifetime of H-PHI/Pt recorded in ethanol in the absence of HCl is shorter (τ_1_ = 2.59 ps) compared to that in the presence of 0.1 M HCl (4.15 ps), suggesting faster electron–hole recombination. Due to strong light scattering by carbon nitride suspension, analysis and fitting of TA decay on the time scale >100 ps is not reliable. Therefore, we investigated excited state dynamics on a longer scale by means of transient photoluminescence decay spectroscopy. All of the samples in both MeCN and ethanol in the presence of HCl (0.1 M) show a photoluminescence with two-step decay, and the kinetic fitting results in two lifetimes, which should be assigned to shallow carrier trapping and deep carrier trapping. For all samples, the lifetime of the trapping state is slightly shorter in ethanol than in MeCN, supporting the role of ethanol as the scavenger of photogenerated holes. Overall, regardless of the solvent, the lifetime of the trapped carriers is the longest for g-C_3_N_4_ and decreases in a series g-C_3_N_4_ > K-PHI > H-PHI > H-PHI/Pt (Table S7). Transition from the covalent structure (g-C_3_N_4_) to the ionic structure (K-PHI) has the most profound impact on the lifetime of trapped carriers. In MeCN, shorter lifetime registered for H-PHI/Pt compared to H-PHI supports our initial assumption that the trapped electrons migrate to Pt. The trapped electrons that have lifetime >0.78 ns (shallow traps) or >4.46 ns (deep traps) migrate to Pt and therefore do not contribute to fluorescence. Similar to fs-TA results, the lifetime of the trapped carriers of H-PHI/Pt recorded in the ethanol in the absence of HCl is shorter compared to that in the presence of 0.1 M HCl (Table S6).

Considering all of the obtained data, we propose the following scheme of processes that occur in the investigated photocatalytic system
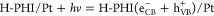
3
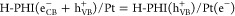
4
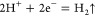
5
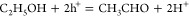
6
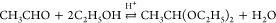
7
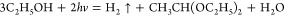
8

Irradiation of the photocatalyst (H-PHI/Pt) with light having photon energy that exceeds its band gap leads to the formation of a bound pair of charges in its volume—an exciton (an electron of the conduction band () and a hole of the valence band ()) (process described by eq 3). After reaching the surface, the electron from the conduction band is transferred to the platinum particles (eq 4) and participates in the reduction of protons (eq 5) and forms hydrogen. At the same time, the hole in the valence band oxidizes ethanol to acetaldehyde (eq 6), the formation of which we recorded experimentally (Figure S8) and which returns back two protons. The resulting acetaldehyde reacts with two ethanol molecules and forms acetal (eq 7). It should be noted that the process of acetal formation from acetaldehyde and ethanol is catalyzed by an acid. Therefore, in our case, acid has a dual role. (i) It increases the photocatalytic activity of K-PHI in the process of hydrogen formation. (ii) It catalyzes the formation of acetal from volatile and toxic acetaldehyde. The overall equation of the process is shown (eq 8), according to which two photons initiate the transformation of three ethanol molecules with the formation of one hydrogen molecule and one acetal molecule.

**Figure 12:**
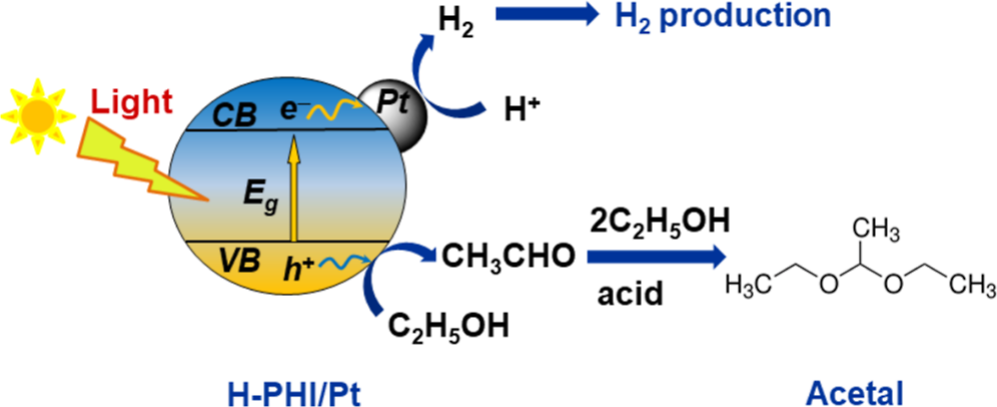
Reaction mechanism diagram of acetal and H_2_ production by H-PHI/Pt surface photocatalytic ethanol conversion.

A reaction mechanism diagram of H_2_ and acetal production is shown in Figure 12. It illustrates the processes that occur on the H-PHI/Pt surface upon irradiation with light.

Conversion of ethanol into acetal and H_2_ by Pt/TiO_2_ without any auxiliary acid was reported.^63,64^ Therein, more acidic surface of TiO_2_ catalyzes nucleophilic attack of ethanol on acetaldehyde and synthesis of acetal, i.e., H^+^ that are released upon ethanol oxidation (eq 6) facilitate acetalization. In other reports,^65−68^ and similar to our own findings, mineral acid (up 1–40 mM) is required to complete acetalization of the in situ generated acetaldehyde. In our case, the stronger basicity of K-PHI is likely the reason for the requirement of an auxiliary acid. It has been reported that upon light irradiation, g-C_3_N_4_ facilitates acetalization of aliphatic carbonyl compounds and benzaldehyde—the photogenerated hole might act as a Lewis acid.^69,70^ Given the abundance of H^+^ in our photocatalytic system, a process described by eq 7 proceeds in the dark.

As can be seen from the figure (Figure S11a), although bulk g-C_3_N_4_ exhibits low activity in the studied process, this material shows relatively high operational stability. In the case of K-PHI (Figure S11b), the rate of hydrogen release during irradiation even slightly increases, which may be due to the partial activation of such a material by protons, the formation of which is possible during the oxidation of ethanol according to the scheme presented above. However, in the case of K-PHI, the hydrogen evolution rate after three cycles is significantly lower than that of H-PHI (Figure S11c). As shown in Figure S11c, the rate of hydrogen formation involving H-PHI gradually decreases during the process, which is consistent with the previously presented results (Figure 11a). Nevertheless, the activity of H-PHI, even after three cycles, significantly exceeds that of the other photocatalysts studied in this work. In particular, after three cycles of 120 min, AQY (410 nm) is 36.7%, 10.2%, and 2.7% for H-PHI, K-PHI, and g-C_3_N_4_, respectively. Notably, irradiation of H-PHI in the absence of acid in the system results in a hydrogen evolution rate during cycling that is almost identical to that observed in the presence of 0.1 M HCl in the system (Figure S11c,d). This result confirms that acid is only needed to activate K-PHI—convert it into H-PHI and facilitate the deposition of platinum nanoparticles. Once H-PHI/Pt is formed, the presence of acid has little effect on the activity and stability of the photocatalytic system.

It should be noted that the rate of acetal formation at the initial stage of irradiation practically coincides with the rate of hydrogen production (Figure 11c), which implies that the AQY of acetal production is close to the AQY of hydrogen production under irradiation at λ = 410 nm. Over time, the yield rate of acetal decreases with respect to hydrogen formation (Figure 11c). According to calculations, this is translated into the decrease of the selectivity of acetal formation from about 90% at the beginning of the reaction to about 75% after the system has been irradiated for 6 h (Figure 11d). A decrease in the selectivity toward acetal can be due to progressively increasing water content, which is formed during the course of the reaction (scheme, eq 7). This results in a shift of the equilibrium toward the formation of acetaldehyde and two ethanol molecules from acetal (see scheme, eq 7).

**Figure 13:**
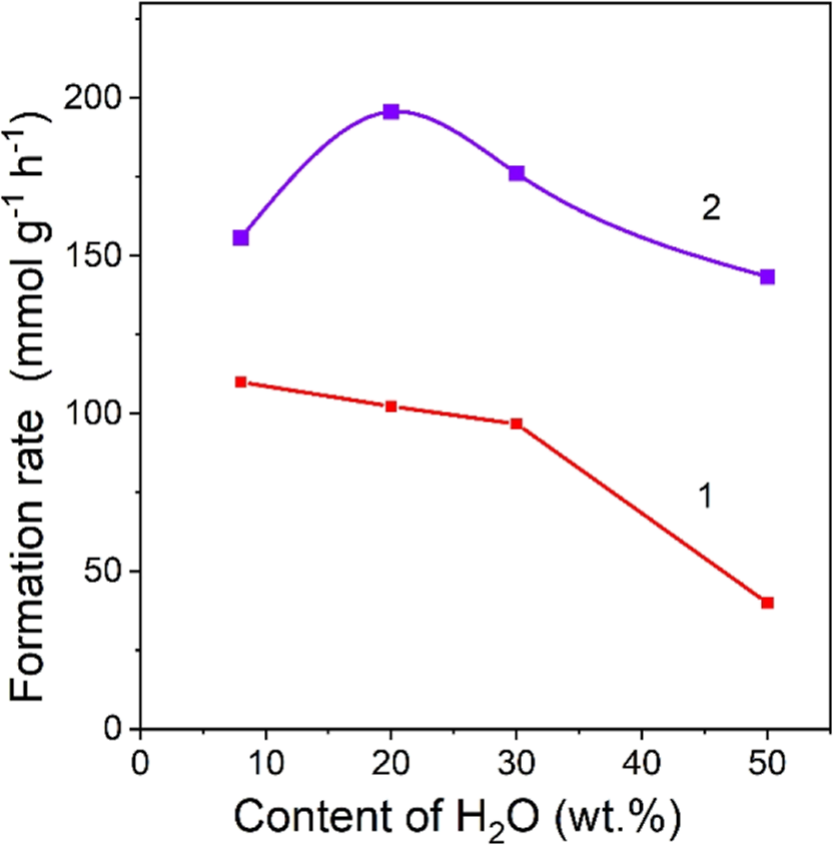
Dependence of the yield rate of acetal (curve 1) and hydrogen (2) on the water content in the system. Conditions: photocatalyst (10 mg), K_2_PtCl_4_ (0.25 μmol), λ = 410 nm (85 mW cm^–2^), *C*(HCl) = 0.01 M, and Ar atmosphere.

To confirm this assumption, we investigated the effect of water concentration in the reaction mixture on the efficiency of photocatalytic production of acetal by using H-PHI/Pt. An increase of water content leads to a gradual decrease of acetal yield rate (Figure 13, curve 1). At the same time, the dependence of hydrogen formation rate on water content in the system shows a maximum at 20 wt % (Figure 13, curve 2). A similar behavior was observed in the synthesis of acetaldehyde and H_2_ from ethanol using Pt/CdS as the photocatalyst.^71^ Therein, however, the highest yield rate of H_2_ was observed when ∼6 M ethanol aqueous solution was used, which corresponds to ∼78 wt % of water in the mixture. Therefore, the decreased yield rate of acetal at higher water content is obviously due to a decrease of the selectivity of the system toward this product and not due to decrease of the activity of the photocatalytic system as a whole, since the activity of hydrogen formation, on the contrary, increases when water content increases from 8 to 20 wt %. Increased activity of the PHI-based system in the hydrogen formation reaction when water content increases from 4 to 20 wt % may be due to a higher concentration of protons–water is a stronger electrolyte compared to ethanol. A decreased activity toward hydrogen formation when water content in the system surpasses 20 wt % could be due to the displacement of ethanol by water on the surface of the photocatalyst,^53^ which leads to the suppression of ethanol oxidation by the photogenerated holes.

**Figure 14:**
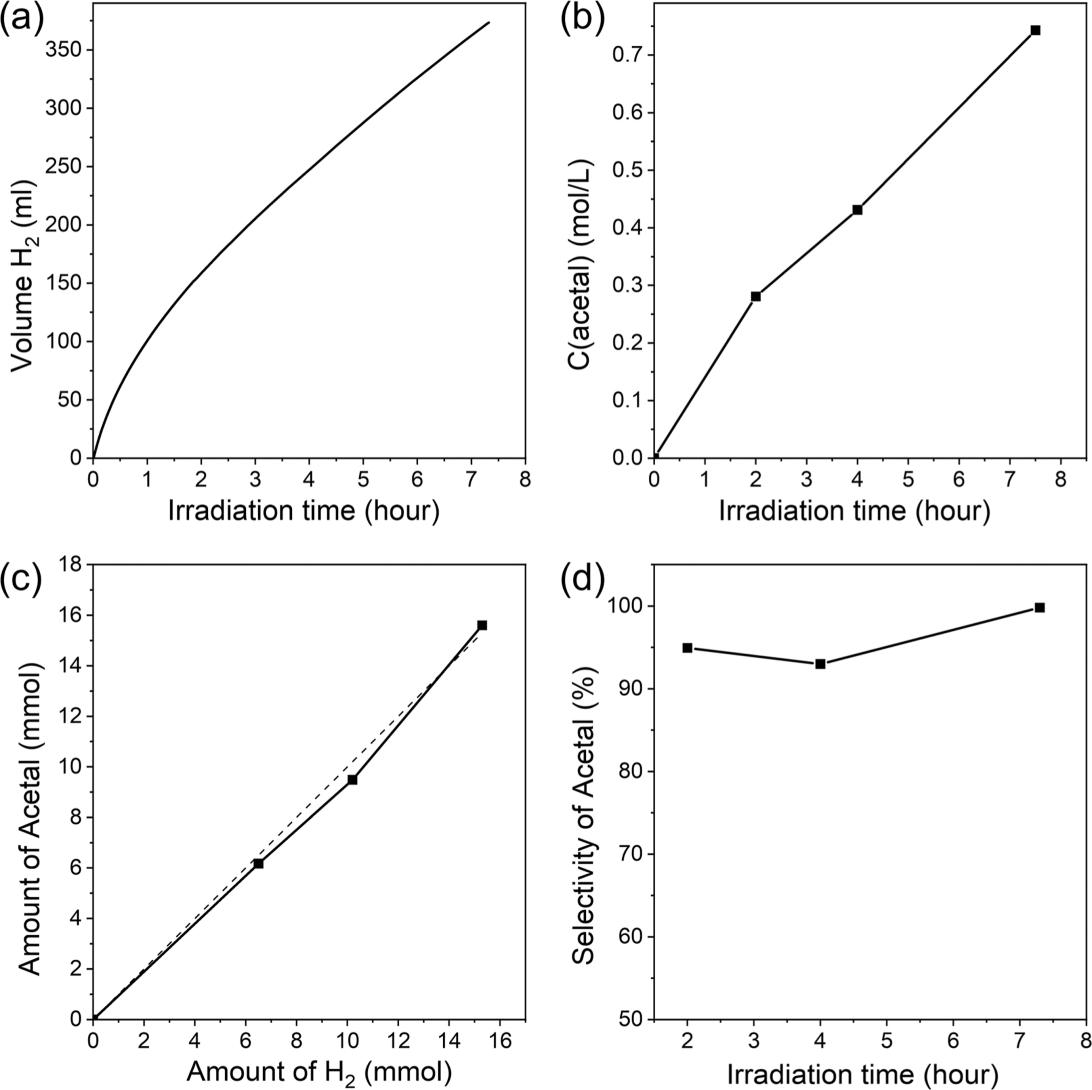
Kinetic curves of simultaneous photocatalytic hydrogen (a) and acetal (b) formation in lab scale reactor. Ratio between the amount of hydrogen and acetal formed during the photoreaction (c). Selectivity of acetal formation in the solution (d). Conditions: photocatalyst—H-PHI/Pt (0.5 wt %) (30 mg), ethanol/water (22 mL, 96:4 wt %), and λ = 410 nm (85 mW cm^–2^).

Next, we investigated the photocatalytic activity of the most active of the obtained materials—H-PHI/Pt that we fixed on a sponge substrate in the simultaneous evolution of hydrogen and synthesis of acetal from ethanol. We used a lab scale reactor, the design of which is given in additional materials (Figure S2). The kinetic curves of hydrogen and acetal production in such a reactor are shown in Figure 14a,b. At the initial stage of irradiation, the rate of hydrogen evolution is quite high and reaches about 217 mmol g^–1^ h^–1^. Irradiation for 2 h leads to a gradual decrease of the efficiency of hydrogen evolution to about 55 mmol g^–1^ h^–1^, which subsequently practically does not change during 5 additional hours of irradiation. A decrease of photocatalytic activity in this case, in addition to the reasons given above, may be partially due to removal of the photocatalyst from the surface of the sponge during irradiation, which, given the low transparency of the sponge, leads to a decrease in the access of light to the photocatalyst in the middle of the sponge. However, the efficiency of such a photocatalytic system, even after a decrease in activity, still remains very high and exceeds the majority of photocatalytic systems reported until now, which operate under visible light irradiation. At the same time, as can be seen from Figure 14c, simultaneously with hydrogen evolution, acetal is formed in the solution. The amount of acetal in this case practically coincides with the amount of hydrogen, which indicates the high selectivity of the acetal formation. It should be noted that no acid additives were introduced into the reactor, in this case, which is important for the practical use of such a system and indicates the critical role of acid only for the deposition of platinum nanoparticles and activation of the photocatalyst. Such an acid-treated photocatalyst obviously contains a sufficient amount of acid groups on its surface to catalyze the process of acetal synthesis. However, the absence of acid in the solution can be the reason for the increased selectivity of acetal formation, since the acid catalyzes the reverse reaction of the decomposition of acetal to the starting ethanol and acetaldehyde.

**Figure 15:**
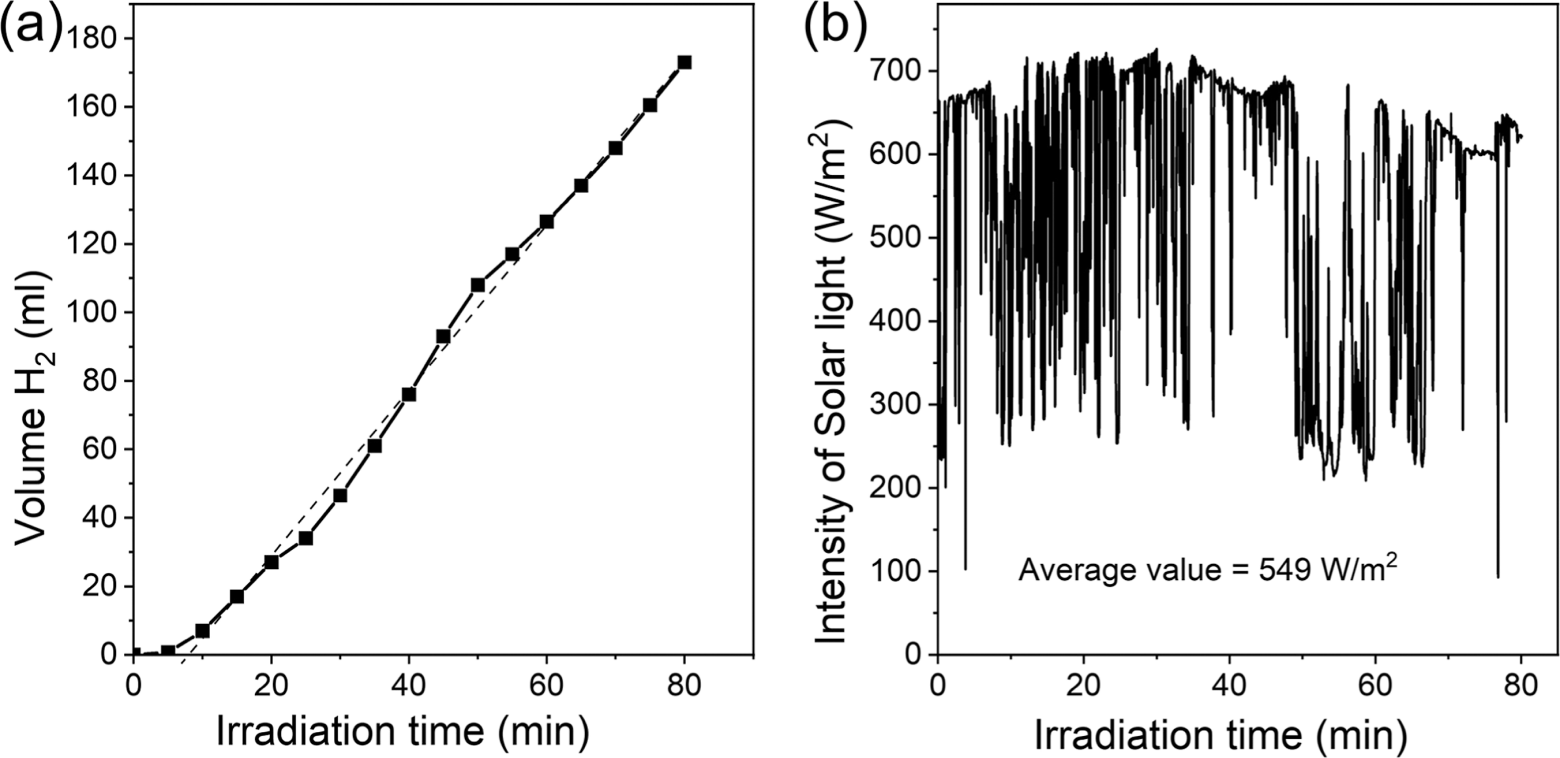
Kinetic curves of photocatalytic hydrogen formation under natural sunlight irradiation of an outdoor 550 cm^2^ reactor (a) and variations of solar illumination intensity during irradiation (b). Conditions: photocatalyst H-PHI/Pt (0.5 wt %) (0.7 g) and ethanol/water (96:4 wt %). This study was conducted on April 8, 2024, starting at 2:00 p.m., Potsdam, Germany. Temperature was about 25–26 °C.

To test the possibility of our photocatalytic system to operate under natural sunlight, a photocatalytic reactor with an irradiation area of 550 cm^2^ was constructed (Figure S3). The kinetic curve of hydrogen formation in such a system under natural sunlight irradiation is shown in Figure 15a. After a short induction period (about 5 min), molecular hydrogen begins to form in the system at a rate of about 144 mL h^–1^ (8.4 mmol g^–1^ h^–1^) (Video 1). At the same time, the average light intensity was about 549 W m^–2^ (Figure 15b), which is lower than expected for the given location and time of the year but is explained by a partially cloudy sky (Figure S4).

## 4 Conclusions

Thus, this paper shows that the treatment of g-C_3_N_4_ in molten salts leads to the formation of K-PHI and an increase in the rate of hydrogen formation from ethanol solutions from 3.0 to 18.2 mmol g^–1^ h^–1^ under visible light irradiation. It was established that the addition of acid to the K-PHI sample leads to a further increase in the activity of the system in the reaction of hydrogen formation from a water–ethanol solution to about 143 mmol g^–1^ h^–1^ under visible light irradiation. This effect of the acid can be explained by the removal of potassium from the structure of carbon nitride, which leads to a change in its ionic structure to a covalent one and an improvement in the migration of photogenerated charges to the surface of the photocatalyst, where redox processes occur. The influence of a number of factors on the activity of the indicated system was determined; in particular, it was shown that the addition of acid allows the studied system to function effectively with a metal cocatalyst content of only 0.05 wt %, which is almost 2 orders of magnitude lower than in most of the systems studied in the literature. The optimal content of platinum in the system, at which the rate of hydrogen formation is maximum, is 0.5 wt %. It was established for the first time that by simply changing the conditions of introducing acid into the system, it is possible to significantly influence the size of Pt (cocatalyst metal) deposition on the H-PHI surface. This approach makes it possible to significantly improve the stability of the photocatalytic system in the reaction of obtaining molecular hydrogen. For the first time, it was shown that the system investigated in this paper works effectively in the presence of air without additional deposition on the surface of the photocatalyst of components blocking the access of air, which in the future can significantly simplify the practical implementation of such photocatalytic systems for obtaining hydrogen. Under the optimal conditions, the AQY of molecular hydrogen production at 410 nm was about 73%, which, as far as we know, is the highest value obtained to date for carbon nitride materials. It was established for the first time that in the case of acid addition to our system, which contains H-PHI photocatalyst, acetal is formed during irradiation with light, which has wide possibilities of practical use and added value.

## Supporting Information Available

Reaction setup, laboratory-scale reactor, outdoor photoreactor, LCA, EPR spectra, chromatograms of liquid phase during irradiation of ethanol solution in the presence of K-PHI and 0.01 M HCl, ^1^H and ^13^C NMR spectra of ethanol solution after irradiation in the presence of H-PHI/Pt, stability tests, table with efficiencies of different photocatalytic system in ethanol conversion and H_2_ production, and fs-TA and transient photoluminescence decay measurements (PDF)
Demonstration of the photoreactor operation under outdoor solar light (MP4)
