# Supplementary material for: Simultaneous Photocatalytic Production of H2 and Acetal from Ethanol with Quantum Efficiency over 73% by Protonated Poly(heptazine imide) under Visible Light
Source: ACS Catal. 2024 Sep 23;14(19):14836–54. doi: 10.1021/acscatal.4c04180 (PMC11459976; doi:10.1021/acscatal.4c04180)
Supplement: Supplementary file 1 — cs4c04180_si_001.pdf [file cs4c04180_si_001.pdf]

Supporting Information

Simultaneous photocatalytic production of H_2 and acetal from ethanol with quantum efficiency over 73% by protonated poly(heptazine imide) under visible light

Vitaliy Shvalagin,[a], Nadezda Tarakina,[a] Bolortuya Badamdorj,[a] Inga-Marie Lahrsen,[b] Eleonora Bargiacchi,[b] Andre Bardow,[b] Ziqi Deng,[c] Wenchao Wang,[c] David Phillips,[c] Zhengxiao Guo,[c] Guigang Zhang,[d] Junwang Tang,[e] Oleksandr Savateev[f],**

[a] *Max Planck Institute of Colloids and Interfaces, Am Mühlenberg 1, 14476 Potsdam, Germany*

[b] *Energy & Process Systems Engineering, Department of Mechanical and Process Engineering, ETH Zurich, Tannenstrasse 3, 8092 Zurich, Switzerland*

[c] *Department of Chemistry, The University of Hong Kong, Hong Kong 999077, SAR, China*

[d] *State Key Laboratory of Photocatalysis on Energy and Environment, College of Chemistry, Fuzhou University, Fujian 350116, China*

[e] *Industrial Catalysis Center, Department of Chemical Engineering, Tsinghua University, Beijing, 100084, China*

[f] *Department of Chemistry, The Chinese University of Hong Kong, Shatin, New Territories, Hong Kong, China*

Corresponding Author

*Email: vitaliy.shvalagin@mpikg.mpg.de (V. Shvalagin)

*Email: oleksandrsavatieiev@cuhk.edu.hk (O. Savateev)

Reaction set up

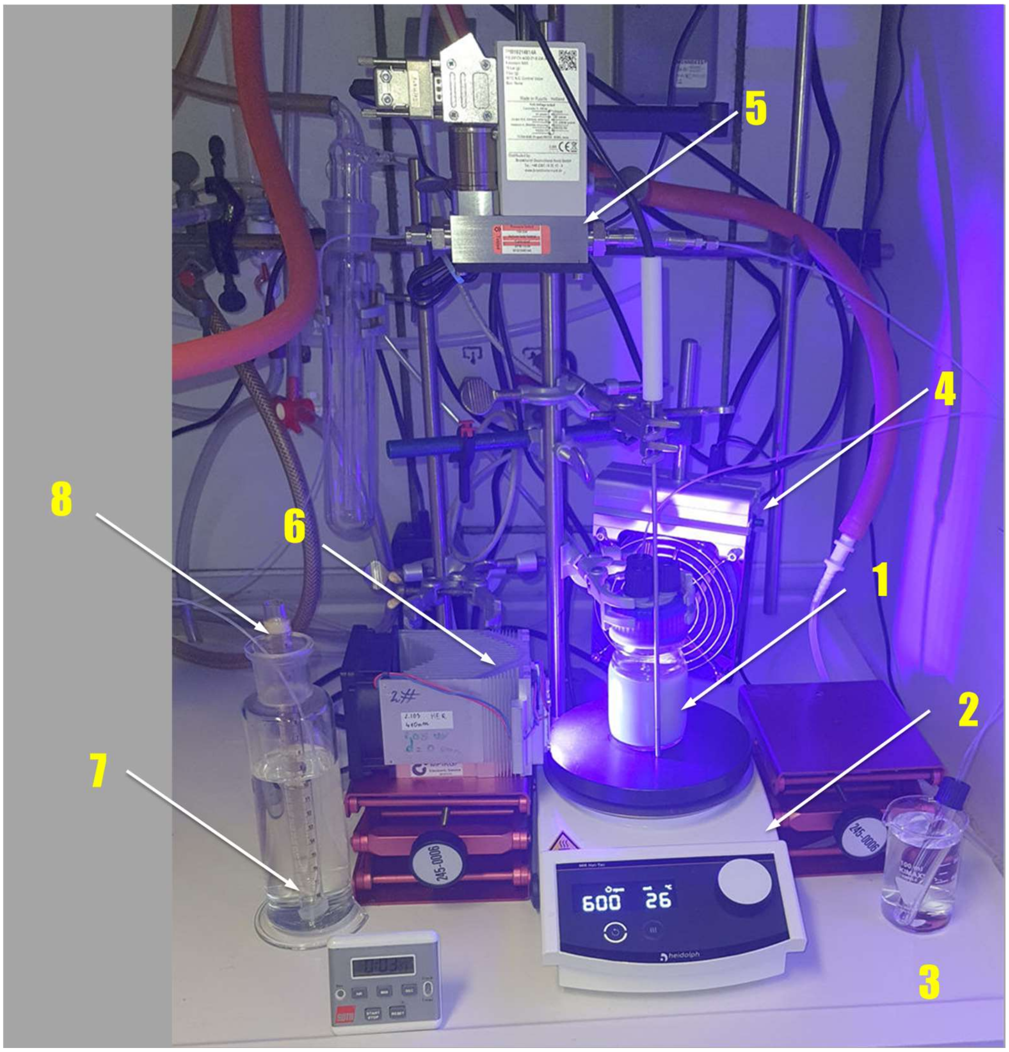

Figure S1. Picture of the reaction set up. (1) Photoreactor 80 or 6 ml, (2) Magnetic stirrer, (3) Trap, (4) Air (fan) cooler, (5) Flowmeter, (6) Light source, (7) Reverse burette filled with water, (8) Septum for sampling gases.

Lab-scale reactor

The photocatalytic activity the most active of the obtained materials - H-PHI/Pt fixed on a sponge substrate was investigated in the reaction of the simultaneous hydrogen evolution from ethanol and the oxidation of ethanol to acetal. A cheap, commercially available sponge made of ecofriendly material consisting of 70% cotton and 30% cellulose was used as the substrate Fig. S2c. A piece of the required

shape was cut from the specified material and a suspension of H-PHI/Pt in ethanol was deposit on it in several stages. After each stage of depositing the photocatalyst suspension, the sponge was dried at 60 °C in air. The total amount of attached photocatalyst was about 30 mg. After final drying, the sponge with the photocatalyst was placed in a lab-scale reactor, the design of which is shown in Fig. S2a, b. Then the reactor was sealed and 22 ml of 96% ethanol was injected into it using a syringe. After that, the reactor was irradiated with visible light (λ=410 nm). The reactor was not deaerated before irradiation. Hydrogen content was measured permanently during irradiation. Periodically during the irradiation, a sample of the liquid phase was taken to determine the content of the ethanol oxidation product - acetal.

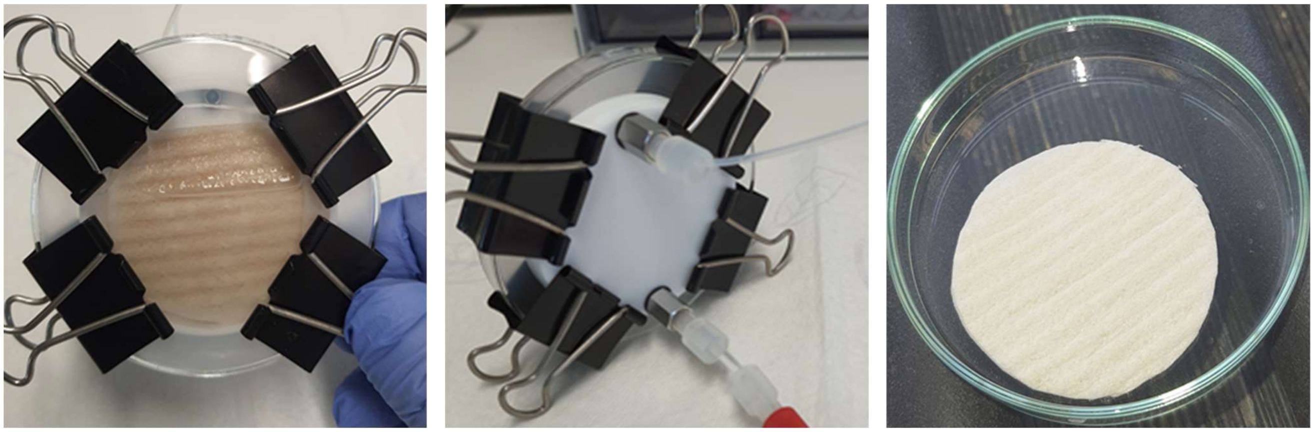

(a) (b) (c)

Figure S2. Photo of a lab-scale photoreactor - front side (a) and back side (b). Photograph of a sponge used as a substrate (c).

Outdoor photoreactor

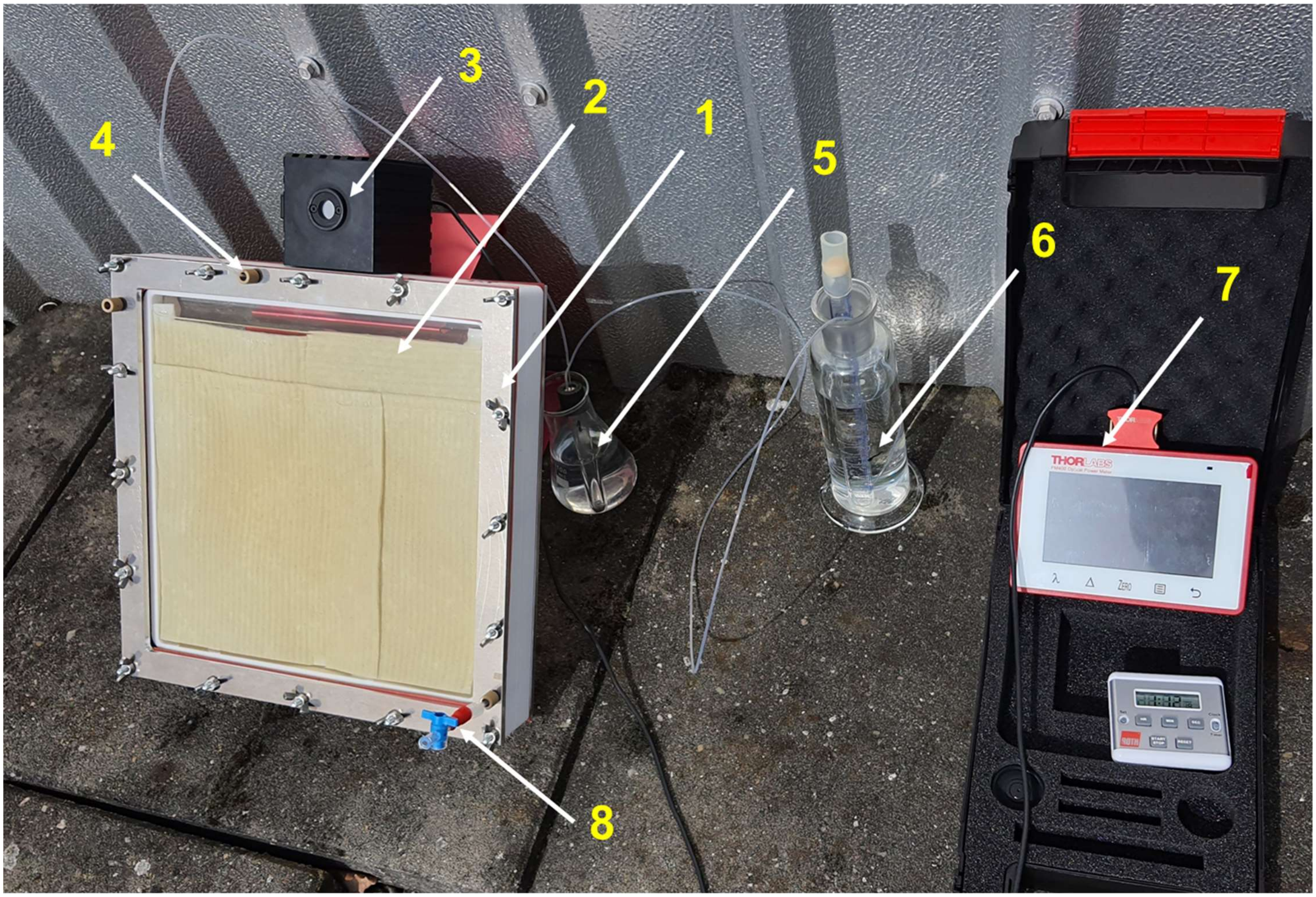

Figure S3. Picture of outdoor reaction set up. (1) Outdoor photoreactor, (2) Sponge with photocatalyst, (3) Photodiode power sensor, (4) Gas outlet, (5) Trap, (6) Reverse burette filled with water with septum for sampling gases, (7) Power meter console, (8) Port for liquid injection and sampling.

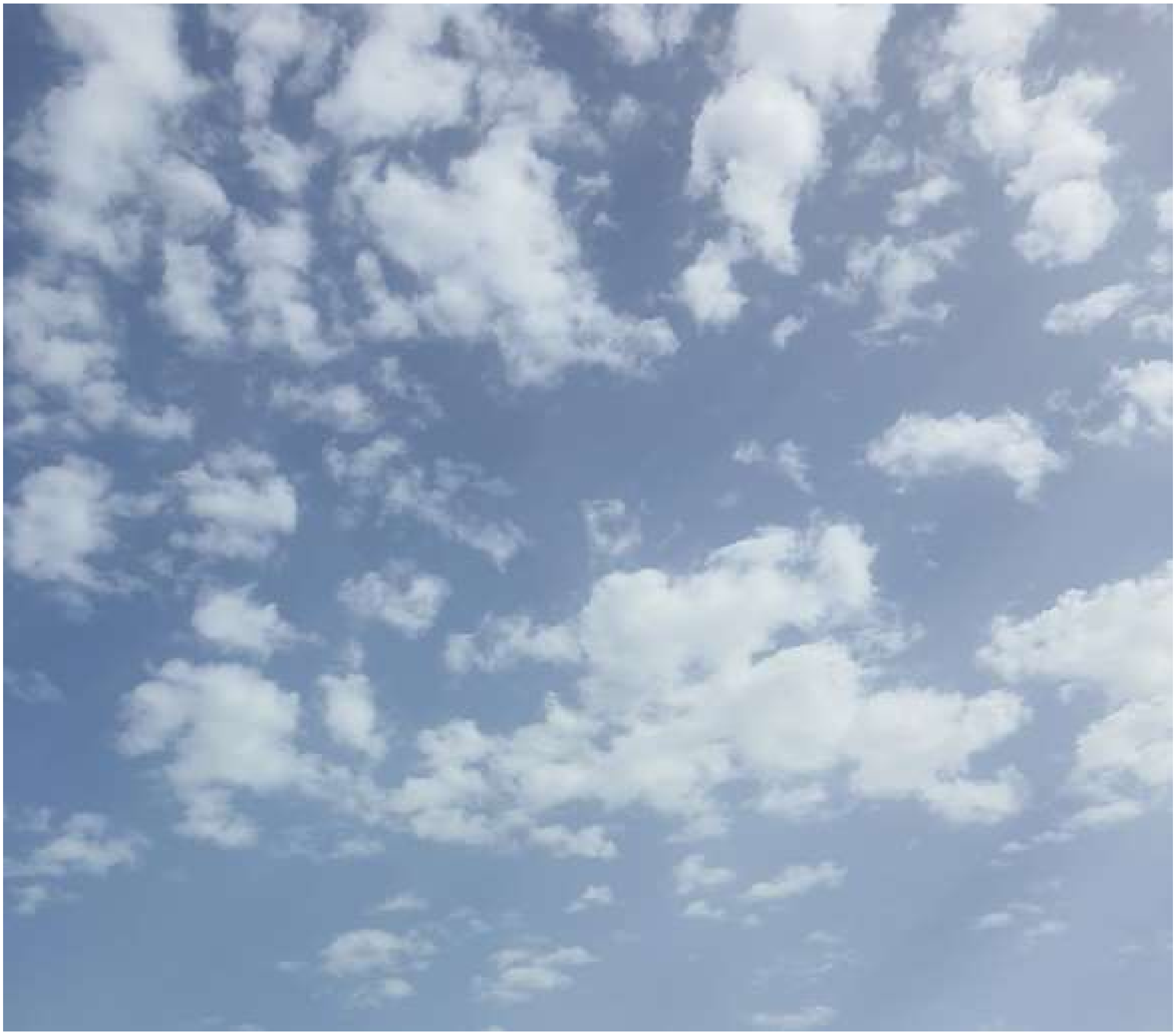

Figure S4 Picture of cloudy sky made during outdoor efficiency measurement (8 of April 2024).

Life cycle assessment

Comparison of the molecular properties of acetal with the benchmark molecules

Based on the boiling point, melting point, and log(P) parameter (Table S1), we assume that acetal can be used as a replacement for MTBE and diethyl ether on a 1:1 molar ratio. We argue that acetal's higher boiling point should be able to cover the same applications as MTBE and diethyl ether. The melting points of the three molecules are all highly below ambient temperature. Moreover, the log(P)

parameters of acetal, MTBE, and diethyl ether are above zero, indicating a higher solubility in fat-like solvents such as n-octanol than in water.

Table S1. Molecular properties of acetal, MTBE, and diethyl ether.

Molecular properties	Acetal	MTBE	Diethyl ether
Boiling point in K[1]	373	328	307
Melting point in K[2]	173.15	164.15	157.15
Log(P)[3]	1.53	1.50	1.37

Calculation of the life cycle inventory

In our life cycle assessment (LCA), we include the entire lifecycle from production to disposal while disregarding the use phase under the assumption of functional equivalence. Our analysis accounts for the greenhouse gas (GHG) emissions resulting from incineration at the end of the product's life, which we calculate using the Doka method.[4] Specifically, we evaluate the incineration impacts for acetal, MTBE, diethyl ether, acetaldehyde, and hydrogen utilizing Doka's framework (Table S2).

Moreover, we consider the biogenic carbon uptake associated with biomass used in ethanol as the feedstock for acetal production in photolysis. It is important to note that in the environmental footprint method 3.0 biogenic carbon uptake is factored with zero since biogenic carbon release at the end of life is equally factored with zero.[5] Since in this work, we include a positive acetal incineration impact at the end of life, we factor biogenic carbon uptake with a negative sign. Thereby, biogenic carbon has a net-zero impact over its full life cycle, but our analysis allows a proper tracking of the actual carbon flows.[6]

Table S2. Incineration impacts of acetal, MTBE, and diethyl ether based on molecular carbon, hydrogen, and oxygen content.

		Acetal	MTBE	Diethyl ether	Acetaldehyde	Hydrogen
atom	molar mass in g/mol	118	88.15	74.12	44.05	2
C	12	6	5	4	2	0
H	1	14	12	10	4	2
O	16	2	1	1	1	0
Impact categories		Doka incineration impacts				
EF v3.0 | acidification | accumulated exceedance (ae)		2.64E-04	1.97E-04	1.66E-04	4.31E-05	1.95E-06
EF v3.0 | climate change | global warming potential (GWP100)		2.10E+00	1.75E+00	1.40E+00	3.06E-01	4.92E-05
EF v3.0 | climate change: biogenic | global warming potential (GWP100)		4.38E-06	3.30E-06	2.76E-06	7.09E-07	2.97E-08
EF v3.0 | climate change: fossil | global warming potential (GWP100)		2.10E+00	1.75E+00	1.40E+00	3.06E-01	4.92E-05

EF v3.0 | climate change: land use and land use change | global warming potential (GWP100)	2.22E-06	1.66E-06	1.40E-06	3.62E-07	1.59E-08
EF v3.0 | ecotoxicity: freshwater | comparative toxic unit for ecosystems (CTUe)	8.71E-02	6.49E-02	5.46E-02	1.42E-02	6.47E-04
EF v3.0 | ecotoxicity: freshwater, inorganics | comparative toxic unit for ecosystems (CTUe)	1.08E-02	8.09E-03	6.79E-03	1.75E-03	7.58E-05
EF v3.0 | ecotoxicity: freshwater, metals | comparative toxic unit for ecosystems (CTUe)	7.43E-02	5.53E-02	4.65E-02	1.21E-02	5.57E-04
EF v3.0 | ecotoxicity: freshwater, organics | comparative toxic unit for ecosystems (CTUe)	2.01E-03	1.51E-03	1.26E-03	3.26E-04	1.40E-05
EF v3.0 | energy resources: non-renewable | abiotic depletion potential (ADP): fossil fuels	7.86E-02	5.89E-02	4.94E-02	1.28E-02	5.59E-04
EF v3.0 | eutrophication: freshwater | fraction of nutrients reaching freshwater end compartment (P)	1.26E-06	9.38E-07	7.90E-07	2.07E-07	9.61E-09
EF v3.0 | eutrophication: marine | fraction of nutrients reaching marine end compartment (N)	1.23E-04	9.18E-05	7.72E-05	2.01E-05	9.09E-07
EF v3.0 | eutrophication: terrestrial | accumulated exceedance (AE)	1.43E-03	1.07E-03	8.97E-04	2.33E-04	1.06E-05
EF v3.0 | human toxicity: carcinogenic | comparative toxic unit for human (CTUh)	2.71E-10	2.02E-10	1.70E-10	4.42E-11	2.01E-12
EF v3.0 | human toxicity: carcinogenic, inorganics | comparative toxic unit for human (CTUh)	0.00E+00	0.00E+00	0.00E+00	0.00E+00	0.00E+00
EF v3.0 | human toxicity: carcinogenic, metals | comparative toxic unit for human (CTUh)	7.84E-12	5.81E-12	4.90E-12	1.29E-12	6.13E-14
EF v3.0 | human toxicity: carcinogenic, organics | comparative toxic unit for human (CTUh)	2.63E-10	1.96E-10	1.65E-10	4.29E-11	1.95E-12
EF v3.0 | human toxicity: non-carcinogenic | comparative toxic unit for human (CTUh)	1.41E-10	1.04E-10	8.76E-11	2.32E-11	1.14E-12
EF v3.0 | human toxicity: non-carcinogenic, inorganics | comparative toxic unit for human (CTUh)	1.02E-10	7.65E-11	6.43E-11	1.67E-11	7.49E-13
EF v3.0 | human toxicity: non-carcinogenic, metals | comparative toxic unit for human (CTUh)	3.03E-11	2.14E-11	1.85E-11	5.20E-12	3.31E-13
EF v3.0 | human toxicity: non-carcinogenic, organics | comparative toxic unit for human (CTUh)	8.93E-12	6.67E-12	5.61E-12	1.46E-12	6.57E-14
EF v3.0 | ionising radiation: human health | human exposure efficiency relative to u235	2.77E-04	2.10E-04	1.75E-04	4.47E-05	1.82E-06
EF v3.0 | land use | soil quality index	2.26E-02	1.70E-02	1.42E-02	3.64E-03	1.49E-04
EF v3.0 | material resources: metals/minerals | abiotic depletion potential (ADP): elements (ultimate reserves)	2.76E-08	2.07E-08	1.74E-08	4.46E-09	1.88E-10
EF v3.0 | ozone depletion | ozone depletion potential (ODP)	6.92E-10	5.18E-10	4.35E-10	1.12E-10	4.89E-12
EF v3.0 | particulate matter formation | impact on human health	2.27E-09	1.69E-09	1.42E-09	3.70E-10	1.67E-11
EF v3.0 | photochemical ozone formation: human health | tropospheric ozone concentration increase	3.82E-04	2.85E-04	2.40E-04	6.24E-05	2.82E-06
EF v3.0 | water use | user deprivation potential (deprivation-weighted water consumption)	1.84E-02	1.38E-02	1.16E-02	3.01E-03	1.36E-04

The process flowsheet (Figure S5. Aspen Plus Flowsheet of acetal production from photolysis.) models the reactions in the photocatalysis for acetal production of the experimental work in this paper. The purge was introduced to improve convergence.

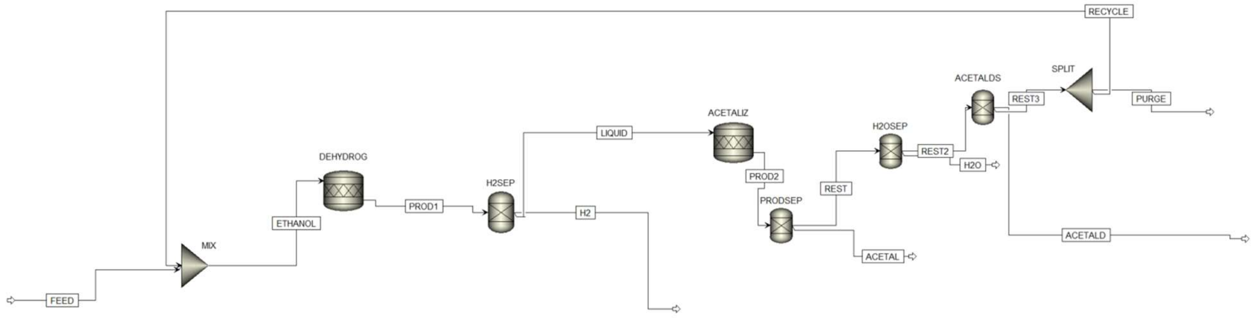

Figure S5. Aspen Plus Flowsheet of acetal production from photolysis.

For the photolysis process in our process flowsheet, we streamline our analysis with the following assumptions:

1. We utilize the NRTL-RK model being the established standard vapor-liquid equilibrium model with available data in Aspen Plus. The data should be validated with experimental data.
2. Only ethanol is considered as feedstock and we neglect water as solvent.
3. Infrastructure and other materials inputs are neglected since their impact is usually small compared to the process itself.[7]
4. Minimal separation energy is considered for separation units, while the reactor energy demands are neglected since we assume that photocatalysis will be solar-driven at industrial scale. We assume heat supply from natural gas. Increasing the separation energy demand by factor 10 leads to an impact increase below 3%, except for non-renewable energy resources (below 5%). Hence, the separation energy demand has a small contribution, and the minimal separation energy demand can be assumed and corrected once experimental data is available.
5. Unreacted ethanol is recycled which is a standard assumption.
6. Ambient conditions are assumed based on the experimental setup in this work.
7. The total reaction balance is represented by two steps:
    a. Dehydrogenation step: $CH_3CH_2OH \rightarrow H_2 + CH_3CHO$
    b. Acetalization step: $2\ CH_3CH_2OH + CH_3CHO \rightarrow (CH_3CH_2O)_2CHCH_3 + H_2O$
    c. Total reaction: $3CH_3CH_2OH \rightarrow (CH_3CH_2O)_2CHCH_3 + H_2 + H_2O$

8. The reaction is modeled in two consecutive stoichiometric reactors based on the ethanol conversion measured in the experiments of this work (2.3 Photocatalytic oxidation of ethanol).
9. Separation processes are categorized as follows:
   a. Separation 1: Hydrogen separation using a component separator.
   b. Separation 2: Acetal product separation using a component separator.
   c. Separation 3: Water separation using a component separator.
   d. Separation 4: Acetaldehyde separation using a component separator.

Assumptions of the life cycle assessment

For the life cycle assessment, we make additional assumptions:

1. In photolysis, we anticipate catalyst recycling at an industrial scale, so catalyst usage is neglected, assuming catalysis recycling as commonly implement in industrial-scale plants.[8]
2. In photolysis, we treat separated water as wastewater.
3. System expansion accounts for hydrogen production and unreacted acetaldehyde in the MTBE and diethyl ether benchmarks. Thus, the functional unit is the production of 1 kg acetal.

We use the Environmental Footprint method 3.1[9] with a cut-off approach and the ecoinvent database, version 3.9,[7] to build the life cycle inventories (Table S3-Table **S4**).

Table S3. Benchmark life cycle inventories from ecoinvent 3.9.

Activity name	Amount	Unit
methyl tert-butyl ether | market for methyl tert-butyl ether | GLO | kilogram | Ecoinvent_CutOff_39	0.75	kilogram
diethyl ether, without water, in 99.95% solution state | diethyl ether production | GLO | kilogram | Ecoinvent_CutOff_39	0.63	kilogram
Benchmark expansion		
acetaldehyde | market for acetaldehyde | GLO | kilogram | Ecoinvent_CutOff_39	0.148	kilogram
hydrogen, gaseous | market for hydrogen, gaseous | GLO | kilogram | Ecoinvent_CutOff_39	0.007	kilogram

Table S4. Photolysis inventory for the production of 1 kg acetal, 0.148 kg of acetaldehyde, and 0.007 kg of hydrogen.

Activity name	Amount	Unit
heat, district or industrial, natural gas | market group for heat, district or industrial, natural gas | GLO | megajoule | Ecoinvent_CutOff_39	0.1044	megajoule
ethanol, without water, in 99.7% solution state, from fermentation | market for ethanol, without water, in 99.7% solution state, from fermentation | GLO | kilogram | Ecoinvent_CutOff_39	1.34	kilogram
wastewater, average | market for wastewater, average | RoW | cubic meter | Ecoinvent_CutOff_39	-0.15	cubic meter
cooling water | cooling water | GLO | unit | Ecoinvent_CutOff_39	0.0009	cubic meter

Extended results

We calculate the environmental impacts of all main impact categories of the Environmental Footprint method 3.1 and normalize the impact scores to the scores of acetal from photolysis (Figure). Benchmark impacts greater than 100% indicate that the benchmark has higher emissions than acetal from photolysis. Benchmark impacts less than 100% indicate that acetal from photolysis has higher impacts than the benchmark. The impacts of diethyl ether production, including system expansion, are especially high in ecotoxicity, ozone depletion, material resources, ionizing radiation, non-renewable energy resources and climate change compared to acetal impacts from photolysis with higher impacts in 10 of 16 impact categories. The impacts of MTBE production, including system expansion, are higher than the impacts of acetal from photolysis in non-renewable energy resources and climate change. The MTBE impacts are lower than the acetal impacts in the remaining impact categories.

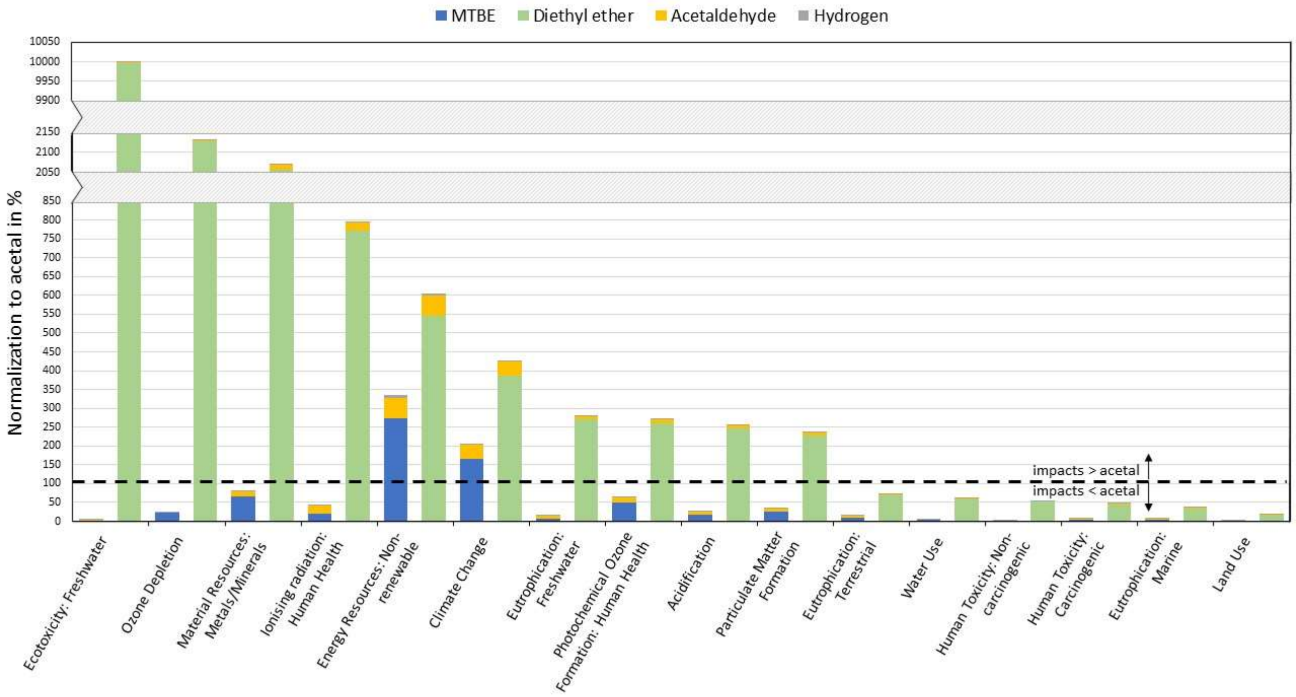

Figure S6. Environmental impacts of MTBE and diethyl ether compared to acetal based on ecoinvent data version 3.9[10] in various impact categories of the Environmental Footprint method 3.1. Benchmark impacts are above 100% when lab-scale acetal production from photolysis results in environmental impact reduction. Acetal production from photolysis includes co-production of acetaldehyde and hydrogen. Benchmark impacts below 100% indicate that acetal production from photolysis has no potential to reduce environmental impacts compared to the benchmark.

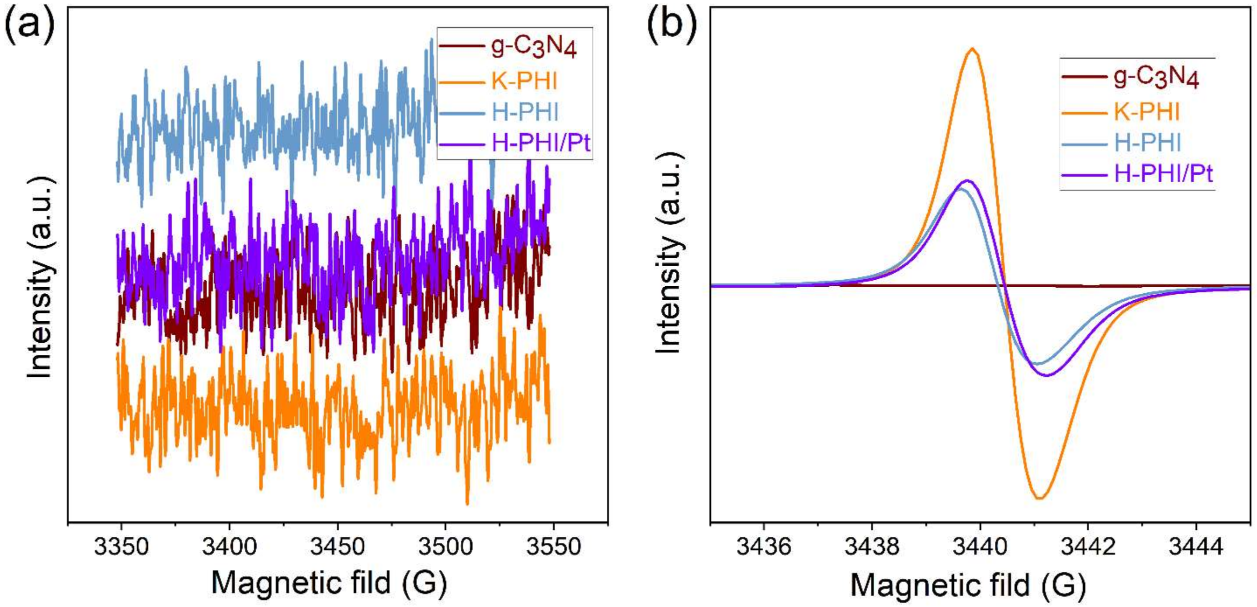

Figure S7. EPR spectra of g-C_3N_4, K-PHI, H-PHI and H-PHI/Pt at room temperature without irradiation (a) and after irradiation for 5 min (b).

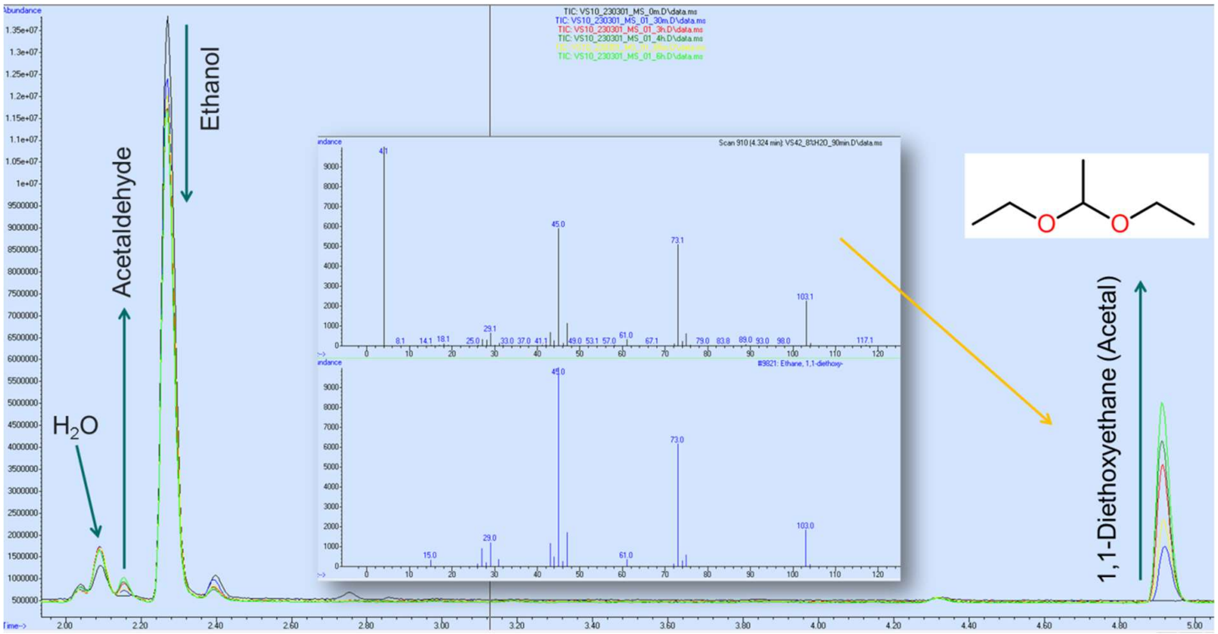

Figure S8. The chromatograms of liquid phase during irradiation of ethanol solution in the presence of K-PHI and 0.01 M HCl. The inset shows the mass spectrum of the obtained product (top) and its comparison with the acetal spectrum from the database (bottom).

1H NMR

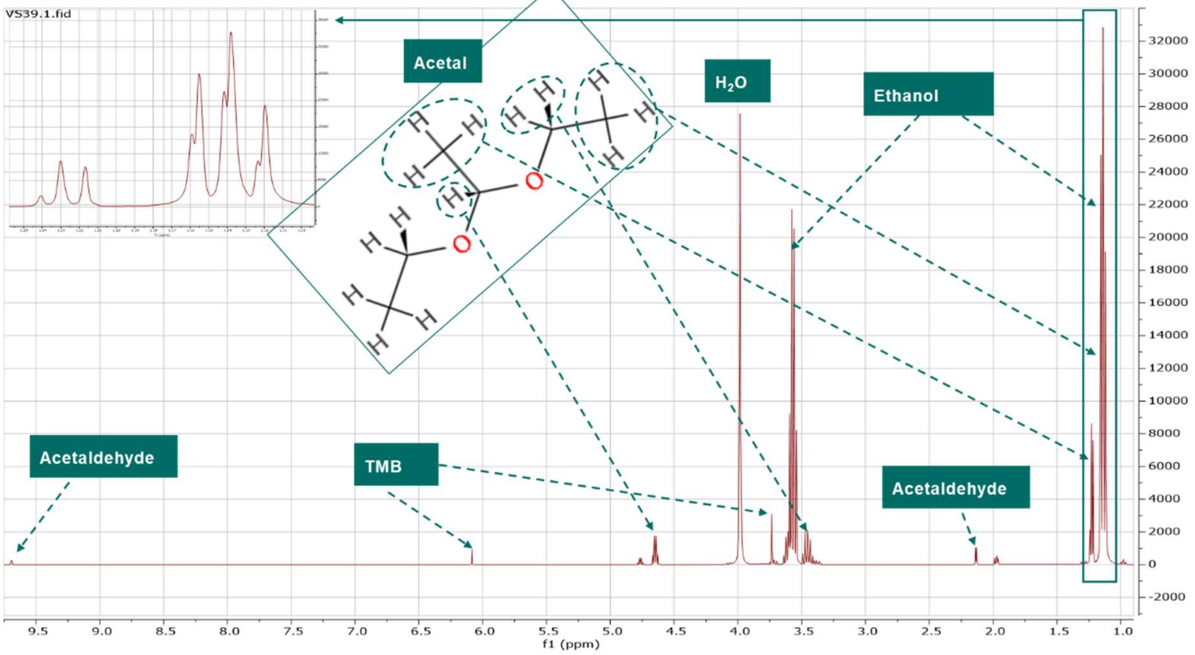

Figure S9. ^{1}H NMR spectrum of the ethanol solution after irradiation with visible light in the presence of H-PHI/Pt. TMB – 1,3,5-trimethoxybenzene.

C13 NMR

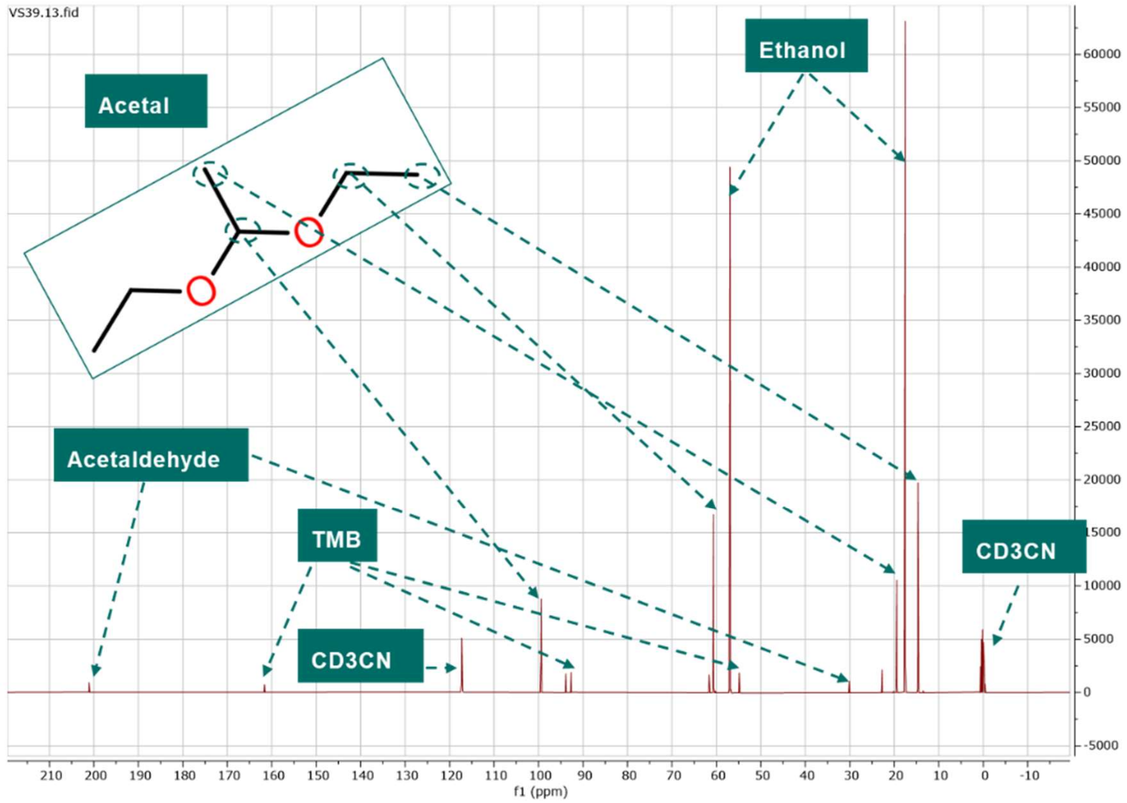

Figure S10. ^{13}C NMR spectrum of the ethanol solution after irradiation with visible light in the presence of H-PHI/Pt. TMB – 1,3,5-trimethoxybenzene.

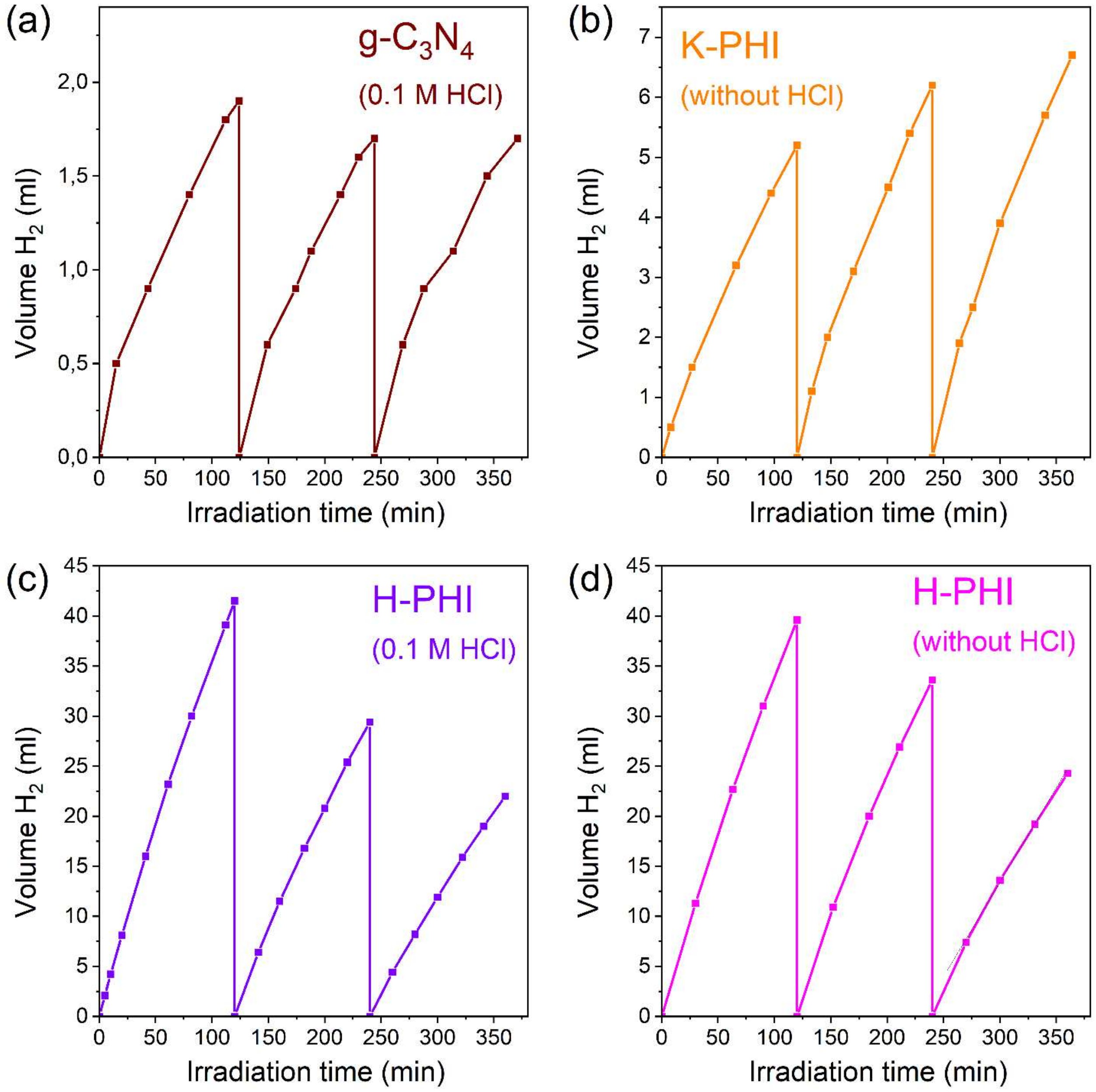

Figure S11. Stability of g-C_3N_4 (a), K-PHI (b), H-PHI with added acid (c) and H-PHI without adding acid (d) in H_2 generation (3 cycles × 120 min). After every 120 min, a catalyst was separated by centrifugation, washed with ethanol and redispersed in a fresh solution of ethanol:water and HCl. Conditions: photocatalyst (10 mg), K_2PtCl_4 (0.25 µmol), ethanol:water (5 mL, 96:4 wt. %), $\lambda = 410$ nm (51 mW cm^{-2}), C(HCl) = 0.1 M, Ar atmosphere. *K-PHI was studied in the absence of acid in the system because the presence of acid would lead to its transformation into H-PHI.

Table S5. Photocatalytic system for H_2 and 1,1-diethoxyethane production from ethanol

Photocatalysts	Cocatalyst	Reactant solution	Light source	H_2 evolution rate (mol g^{-1} h^{-1}), and AQY	Main organic product, and rate (mol g^{-1} h^{-1})	Ref.
H-PHI	0.5 wt% Pt	Ethanol	50 W LED 410 nm, 0.085 W cm^{-2}	0.142 (0.075 after 6 hours), 73% 410 nm	1,1-diethoxyethane 0.118 (0.054 after 6 hours)	This work
CdS	10wt% Ti3C2Tx	Ethanol	Xe lamp > 420 nm	0.0171	1,1-diethoxyethane, 0.0047	11
CdS	7wt% Ni-MoS2	Ethanol	Xe lamp > 420 nm	-	1,1-diethoxyethane, 0.0174	12
TiO_2	1wt% Pd	Ethanol	Xe lamp 300-800 nm, 0.8 W cm^{-2}	0.0515, 5.3% 365 nm	1,1-diethoxyethane, 0.0077	13
TiO_2	1wt% Pd	Ethanol	High-pressure Hg lamp	0.0350	1,1-diethoxyethane, 0.0262	14
TiO_2	1wt% Pd	Ethanol	High-pressure Hg lamp	-	1,1-diethoxyethane, 0.0528	15
TiO_2	1wt% Pd	Ethanol	High-pressure Hg lamp	-	1,1-diethoxyethane, 0.0364	16

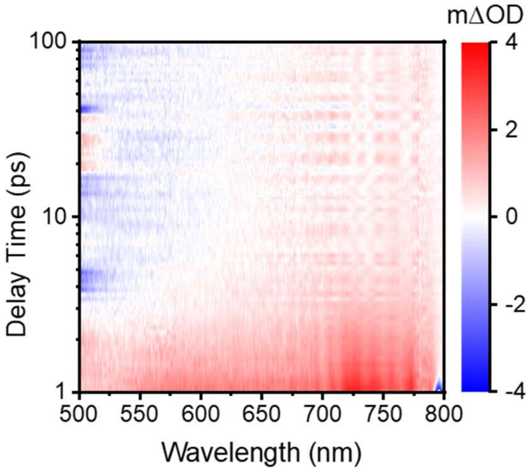

(a)

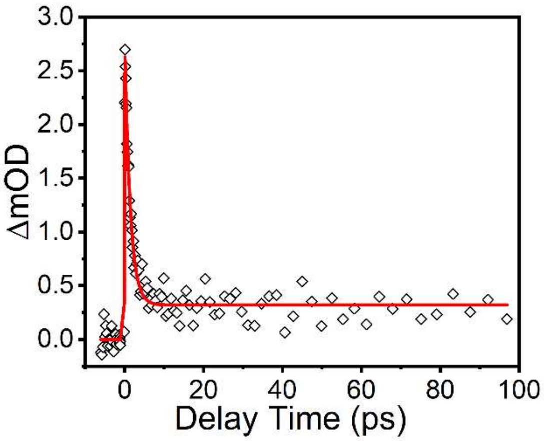

(b)

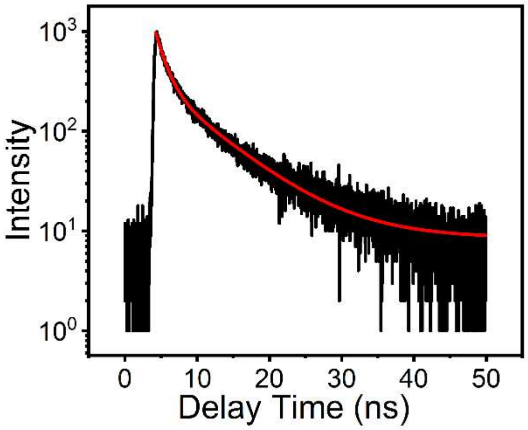

(c)

Figure S12. Time-dependent 3D contour plots of fs-TAS of g-C_3N_4 acetonitrile suspension (a), single-wavelength (700 nm) kinetic extracted from the recorded 3D matrices (b) and transient photoluminescence decay spectroscopy measurements of g-C_3N_4 in acetonitrile at 450 nm (c).

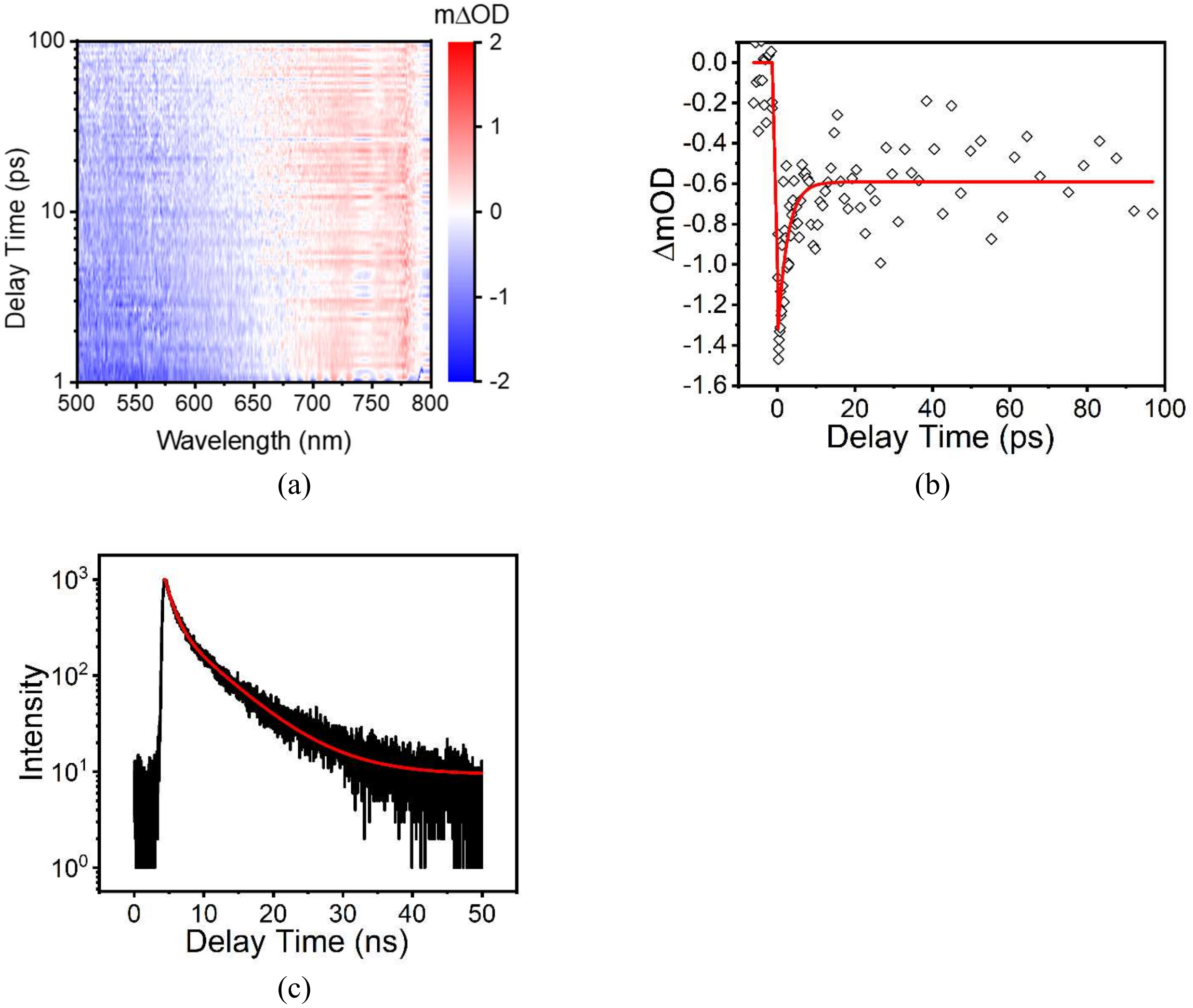

Figure S13. Time-dependent 3D contour plots of fs-TAS of g-C_3N_4 ethanol suspension (a), single-wavelength (525 nm) kinetic extracted from the recorded 3D matrices (b) and transient photoluminescence decay spectroscopy measurements of g-C_3N_4 in ethanol at 450 nm (c).

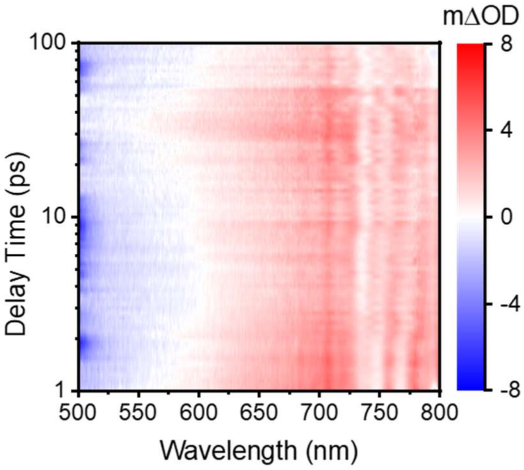

(a)

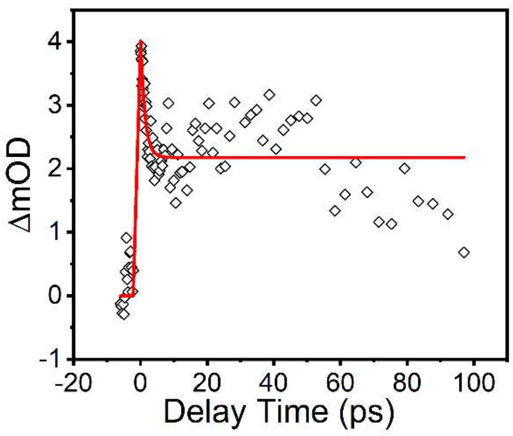

(b)

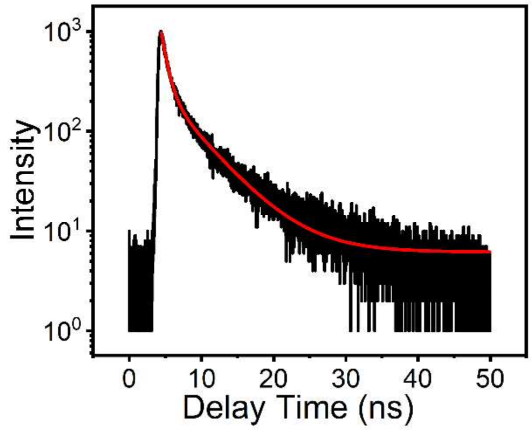

(c)

Figure S14. Time-dependent 3D contour plots of fs-TAS of K-PHI acetonitrile suspension (a), single-wavelength (700 nm) kinetic extracted from the recorded 3D matrices (b) and transient photoluminescence decay spectroscopy measurements of K-PHI in acetonitrile at 450 nm (c).

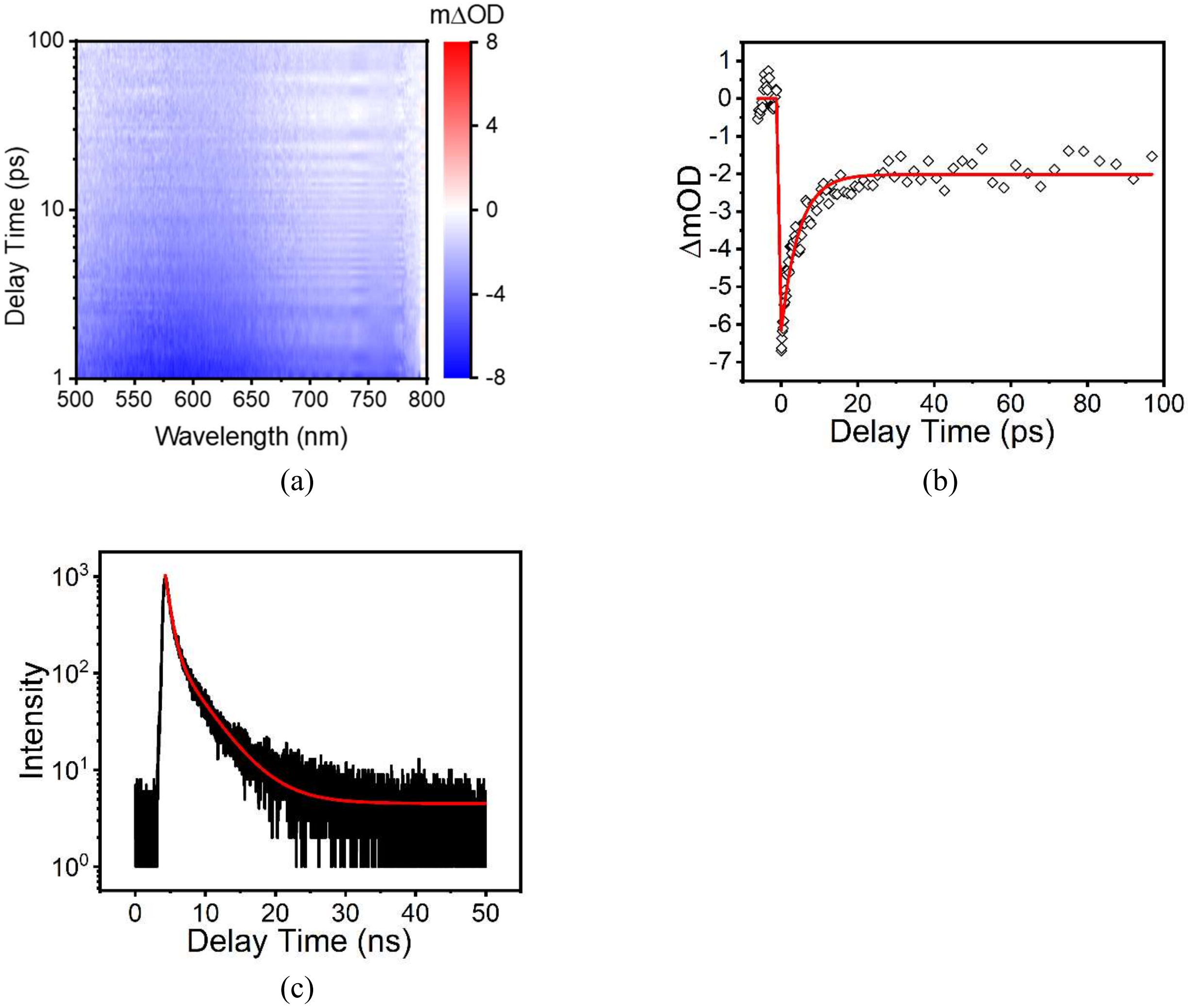

Figure S15. Time-dependent 3D contour plots of fs-TAS of K-PHI ethanol suspension (a), single-wavelength (560 nm) kinetic extracted from the recorded 3D matrices (b) and transient photoluminescence decay spectroscopy measurements of K-PHI in ethanol at 450 nm (c).

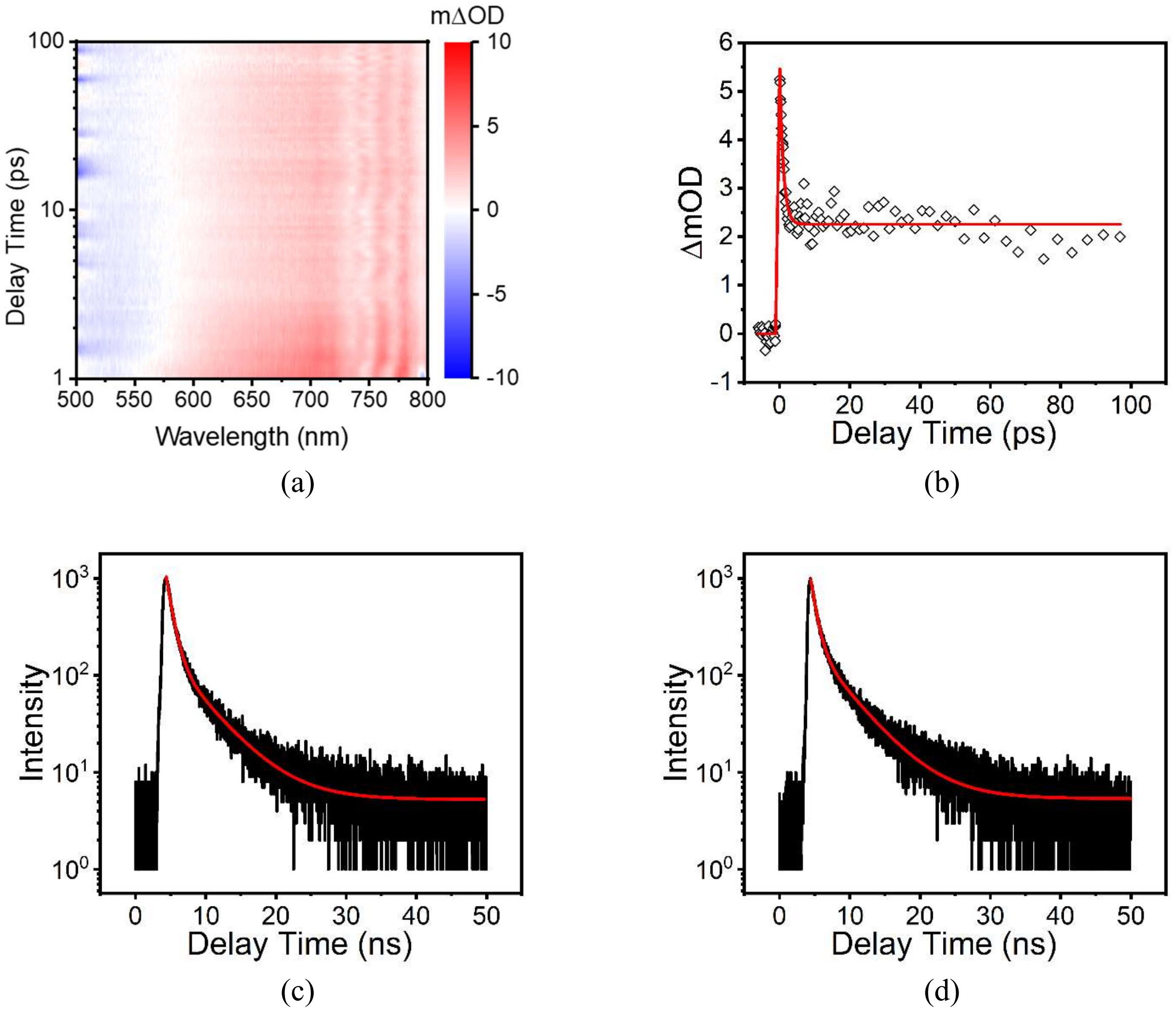

Figure S16. Time-dependent 3D contour plots of fs-TAS of H-PHI acetonitrile suspension (a), single-wavelength (700 nm) kinetic extracted from the recorded 3D matrices (b), transient photoluminescence decay spectroscopy measurements of H-PHI in acetonitrile at 450 nm (c) and 500 nm (d).

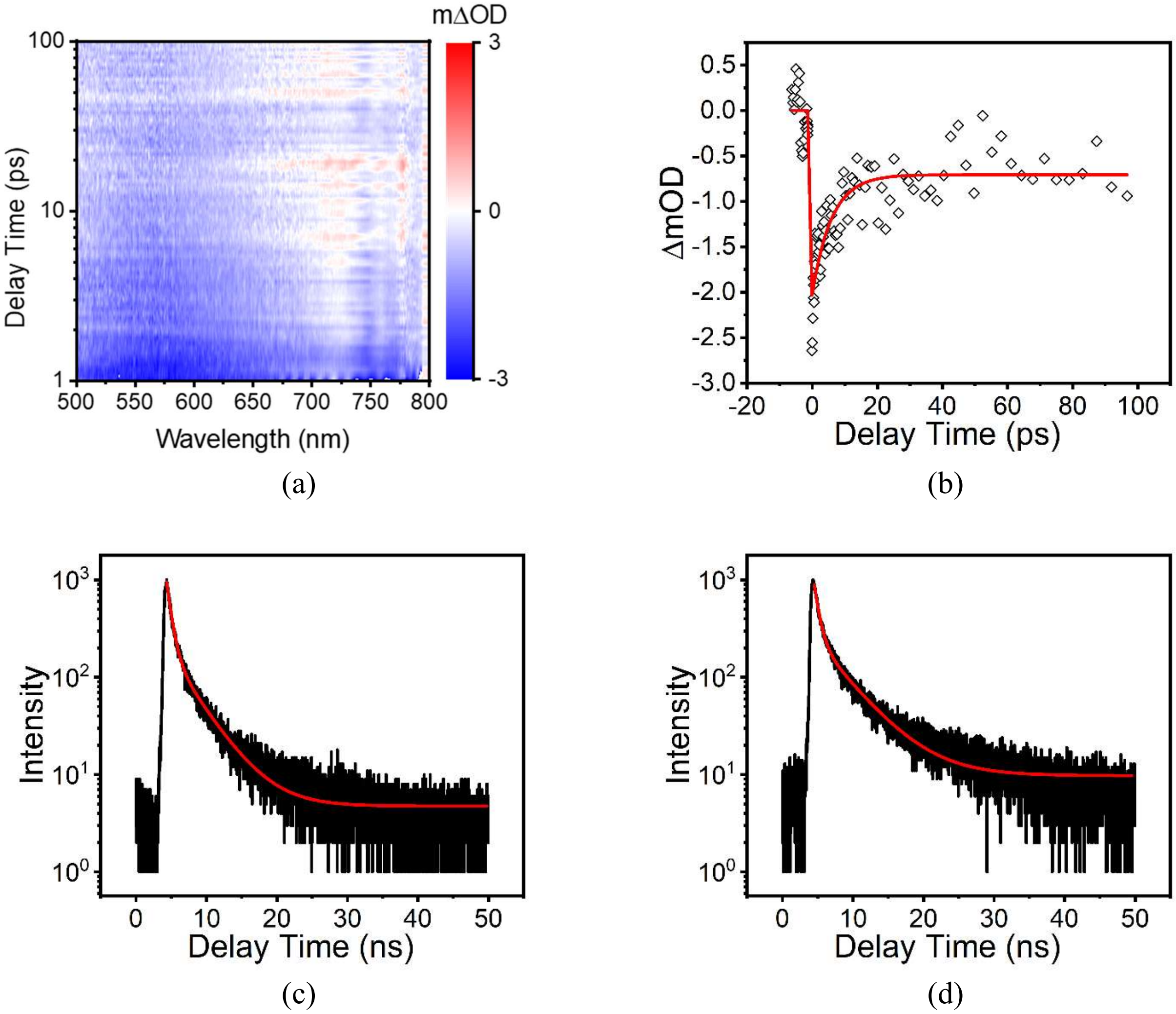

Figure S17. Time-dependent 3D contour plots of fs-TAS of H-PHI ethanol suspension (a), single-wavelength (560 nm) kinetic extracted from the recorded 3D matrices (b), transient photoluminescence decay spectroscopy measurements of H-PHI in ethanol at 450 nm (c) and 500 nm (d).

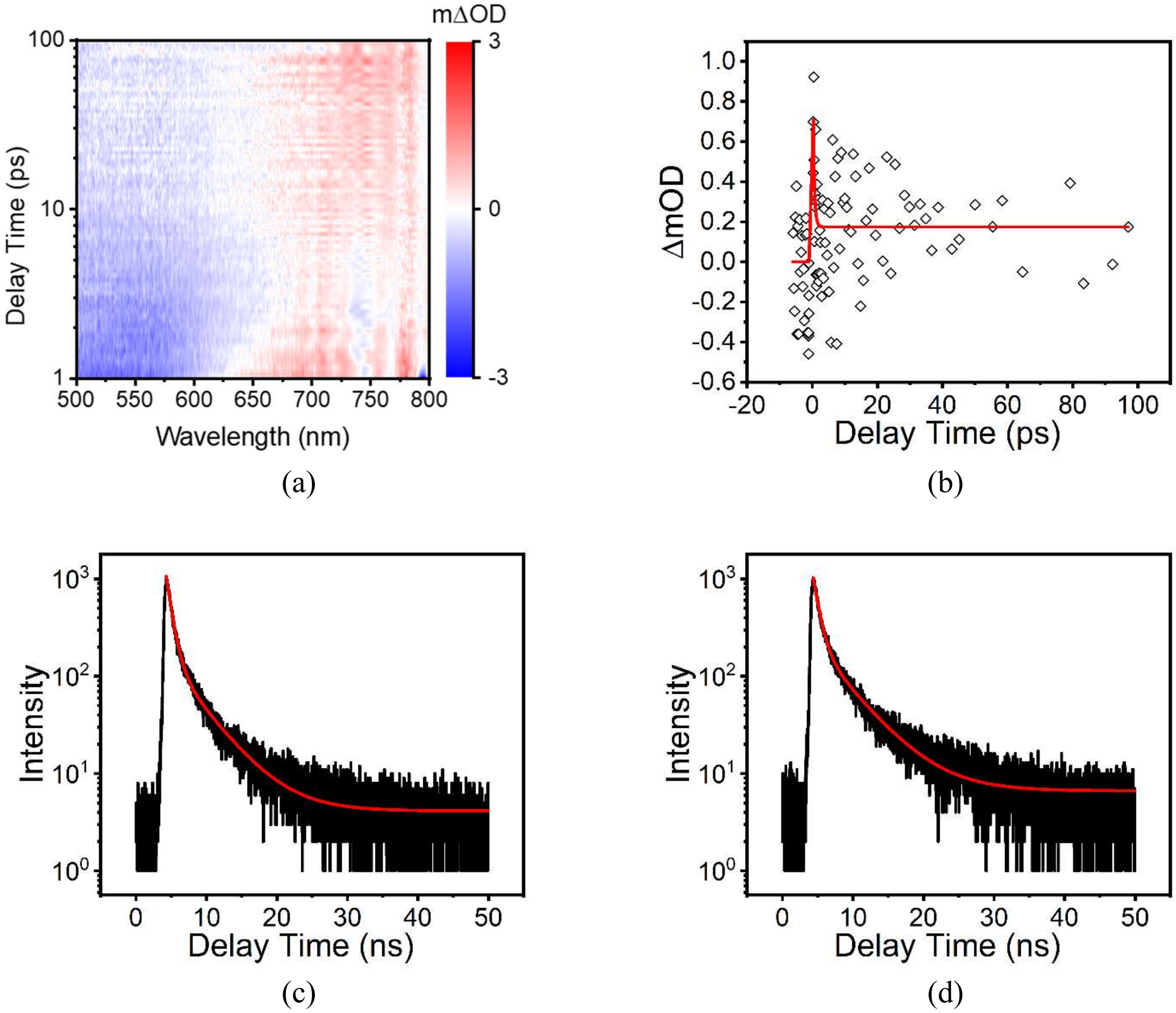

Figure S18. Time-dependent 3D contour plots of fs-TAS of H-PHI/Pt acetonitrile suspension (a), single-wavelength (700 nm) kinetic extracted from the recorded 3D matrices (b), transient photoluminescence decay spectroscopy measurements of H-PHI/Pt in acetonitrile at 450 nm (c) and 500 nm (d).

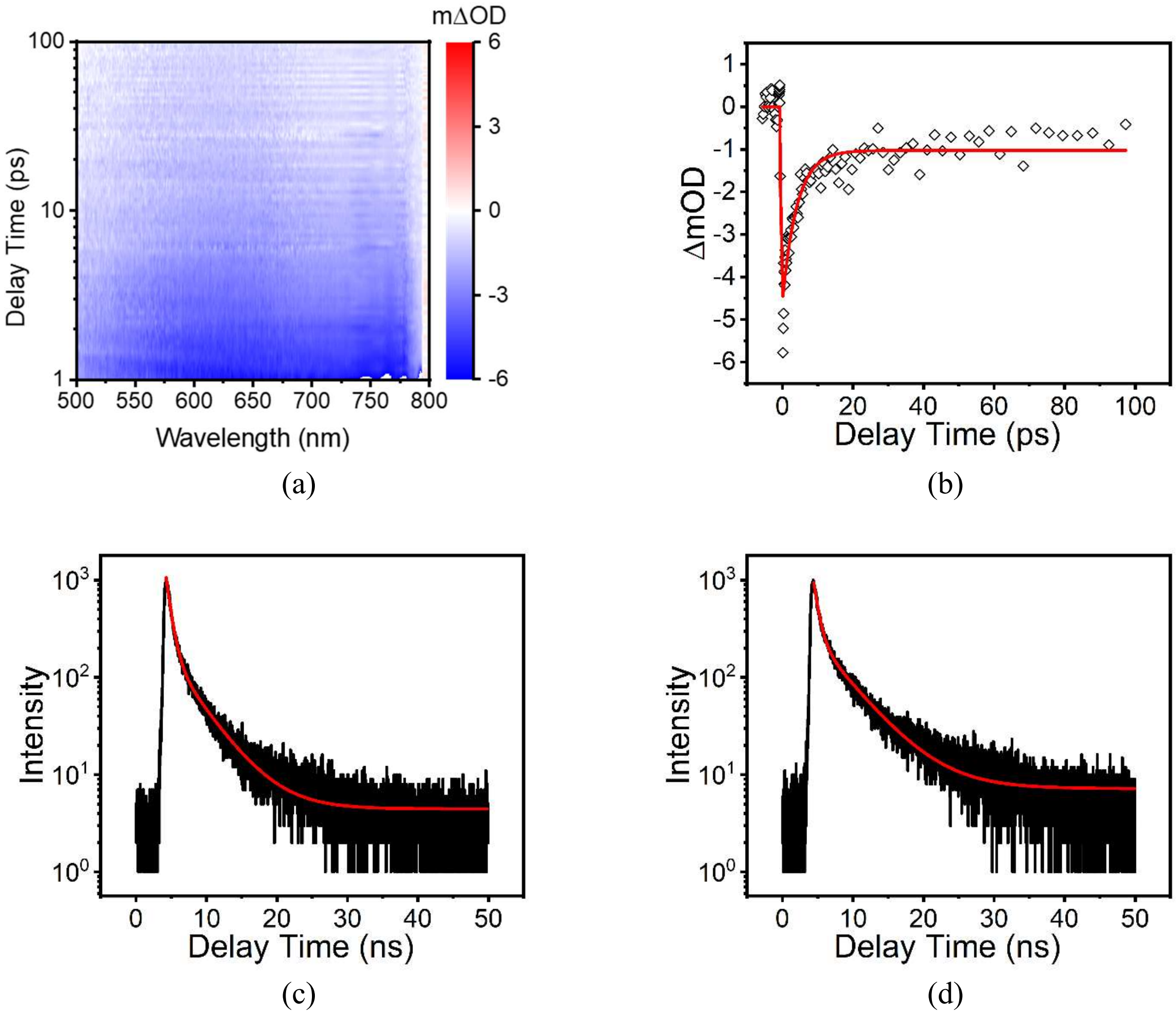

Figure S18. Time-dependent 3D contour plots of fs-TAS of H-PHI/Pt ethanol suspension (a), single-wavelength (560 nm) kinetic extracted from the recorded 3D matrices (b), transient photoluminescence decay spectroscopy measurements of H-PHI/Pt in ethanol at 450 nm (c) and 500 nm (d).

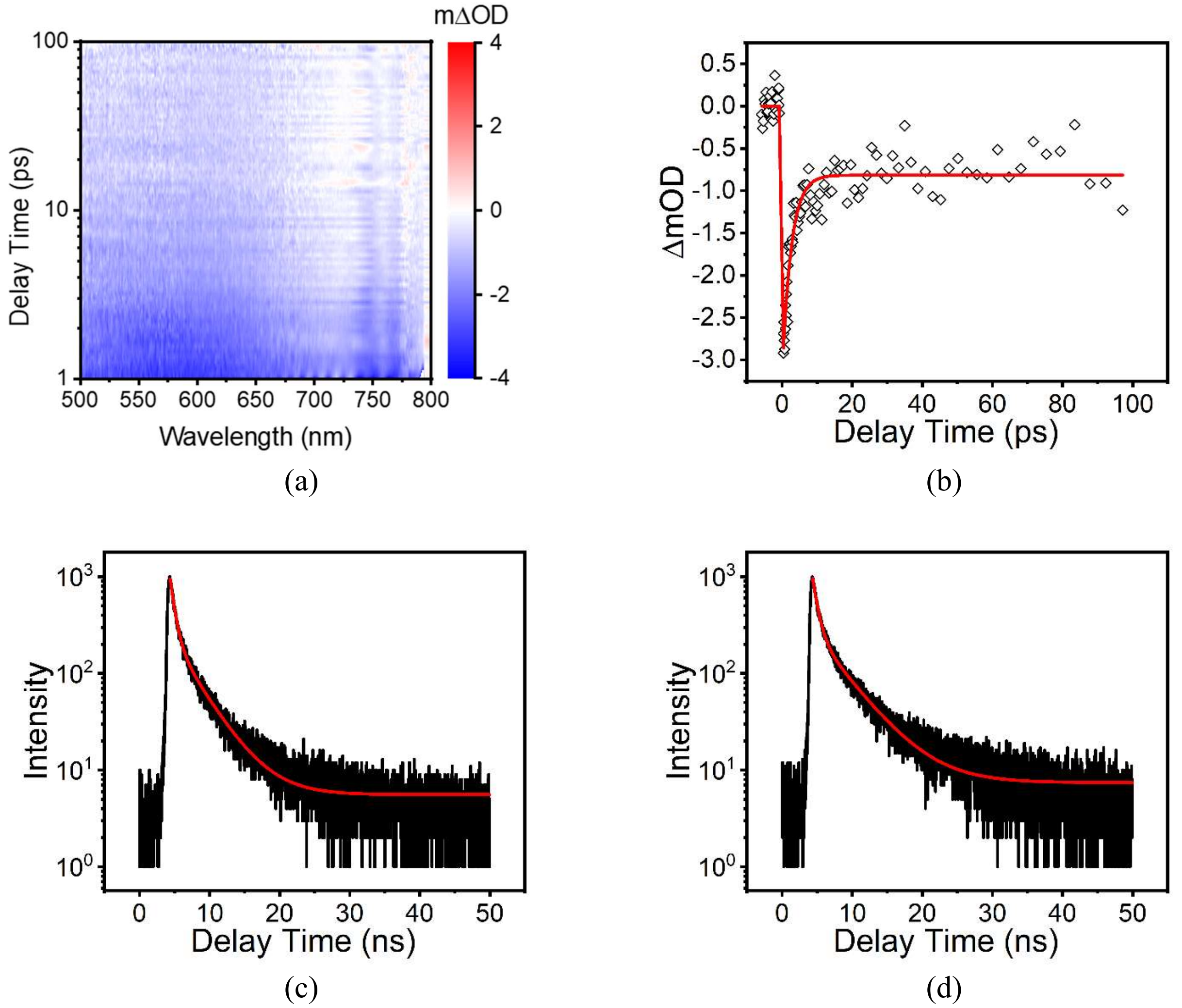

Figure S20. Time-dependent 3D contour plots of fs-TAS of H-PHI/Pt ethanol suspension (a), single-wavelength (560 nm) kinetic extracted from the recorded 3D matrices (b), transient photoluminescence decay spectroscopy measurements of H-PHI/Pt in ethanol at 450 nm (c) and 500 nm (d). In the absence of HCl.

Table S6. Results of transient absorption spectroscopy measurements.

**Sample**	**Solvent**	**τ, ps**
g-C_3N_4	MeCN, 0.1M HCl	1.41
K-PHI	MeCN, 0.1M HCl	1.31
H-PHI	MeCN, 0.1M HCl	1.05
H-PHI/Pt	MeCN, 0.1M HCl	0.41
g-C_3N_4	EtOH, 0.1M HCl	2.56

K-PHI	EtOH, 0.1M HCl	4.65
H-PHI	EtOH, 0.1M HCl	4.24
H-PHI/Pt	EtOH, 0.1M HCl	4.15
H-PHI/Pt	EtOH	2.59

Table S7. Results of transient photoluminescence decay spectroscopy measurements.

**Sample**	**Solvent**	**A_1**	**τ_1, ns**	**A_2**	**τ_2, ns**	**$\langle\tau\rangle$,[a] ns**
g-C_3N_4	MeCN, 0.1M HCl	636.19248	1.31916	280.88655	7.23939	5.51
K-PHI	MeCN, 0.1M HCl	892.50855	0.83217	249.20232	5.10535	3.53
H-PHI	MeCN, 0.1M HCl	949.08805	0.87498	157.81556	4.85895	2.79
H-PHI/Pt	MeCN, 0.1M HCl	1026.76391	0.78496	150.92842	4.46112	2.46
g-C_3N_4	EtOH, 0.1M HCl	670.60512	1.20754	342.64996	6.47787	5.07
K-PHI	EtOH, 0.1M HCl	949.54625	0.72563	187.19173	3.99969	2.43
H-PHI	EtOH, 0.1M HCl	917.88925	0.67064	189.57749	3.81963	2.37

H-PHI/Pt	EtOH, 0.1M HCl	1005.35309	0.70429	176.01909	4.08644	2.41
H-PHI/Pt	EtOH	21.34925	0.65512	121.64246	3.62276	3.53

[a] Average lifetime was calculated according to the equation:

$$< t >= \frac{A_1\tau_1^2 + A_2\tau_2^2}{A_1\tau_1 + A_2\tau_2}$$

References

(1) NIST Chemistry WebBook. NIST Office of Data and Informatics, "NIST Chemistry WebBook." Accessed: Apr. 03, 2024. [Online]. Available: https://webbook.nist.gov/chemistry/.

(2) GESTIS-Stoffdatenbank. "GESTIS-Stoffdatenbank." Accessed: Mar. 11, 2024. [Online]. Available: https://gestis.dguv.de/data?name=020050.

(3) Klamt, A. *COSMO-RS: From Quantum Chemistry to Fluid Phase Thermodynamic and Drug Design*; Elsevier: Amsterdam, 2005.

(4) Doka, G. *Life Cycle Inventories of Waste Treatment Services*; Swiss Centre for Life Cycle Inventories, 2003.

(5) Sonderegger, T.; Stoikou, N. Implementation of Life Cycle Impact Assessment Methods in the Ecoinvent Database v3. 9 and v3. 9.1. Implementation of Life Cycle Impact Assessment Methods in the Ecoinvent Database v3. 9 and v3. 9.1, Zürich, Switzerland, 2022.

(6) Pawelzik, P.; Carus, M.; Hotchkiss, J.; Narayan, R.; Selke, S.; Wellisch, M.; Weiss, M.; Wicke, B.; Patel, M. K. Critical aspects in the life cycle assessment (LCA) of bio-based materials – Reviewing methodologies and deriving recommendations. *Resour. Conserv. Recycl.* **2013**, *73*, 211-228. DOI: https://doi.org/10.1016/j.resconrec.2013.02.006

(7) Althaus, H.-J.; Hischier, R.; Osses, M. Life Cycle Inventories of Chemicals. ecoinvent report No. 8, v2.0,. Swiss Centre for Life Cycle Inventories, 2007.

(8) Piccinno, F.; Hischier, R.; Seeger, S.; Som, C. From laboratory to industrial scale: a scale-up framework for chemical processes in life cycle assessment studies. *J. Clean. Prod.* **2016**, *135*, 1085-1097. DOI: https://doi.org/10.1016/j.jclepro.2016.06.164

(9) Andreasi, B. S.; Biganzoli, F.; Ferrara, N.; Amadei, A.; Valente, A.; Sala, S.; Ardente, F. *Updated characterisation and normalisation factors for the Environmental Footprint 3.1 method*; Publications Office of the European Union, Luxembourg, 2023.
(10) *Ecoinvent Association, "ecoinvent data v3.9, cut-off." https://ecoinvent.org/* 2023. (accessed 2023-11-21).
(11) Li, J.-Y.; Li, Y.-H.; Zhang, F.; Tang, Z.-R.; Xu, Y.-J. Visible-light-driven integrated organic synthesis and hydrogen evolution over 1D/2D CdS-Ti3C2Tx MXene composites. *Appl. Catal. B: Environ.* **2020**, *269*, 118783. DOI: https://doi.org/10.1016/j.apcatb.2020.118783
(12) Chao, Y.; Zhang, W.; Wu, X.; Gong, N.; Bi, Z.; Li, Y.; Zheng, J.; Zhu, Z.; Tan, Y. Visible-Light Direct Conversion of Ethanol to 1,1-Diethoxyethane and Hydrogen over a Non-Precious Metal Photocatalyst. *Chem. Eur. J.* **2019**, *25* (1), 189-194. DOI: https://doi.org/10.1002/chem.201804664
(13) Weng, B.; Quan, Q.; Xu, Y.-J. Decorating geometry- and size-controlled sub-20 nm Pd nanocubes onto 2D TiO2 nanosheets for simultaneous H2 evolution and 1,1-diethoxyethane production. *J. Mater. Chem. A* **2016**, *4* (47), 18366-18377, 10.1039/C6TA07853B.
(14) Zhang, H.; Zhu, Z.; Wu, Y.; Zhao, T.; Li, L. TiO2-photocatalytic acceptorless dehydrogenation coupling of primary alkyl alcohols into acetals. *Green Chem.* **2014**, *16* (9), 4076-4080, 10.1039/C4GC00413B.
(15) Zhang, H.; Wu, Y.; Li, L.; Zhu, Z. Photocatalytic Direct Conversion of Ethanol to 1,1-Diethoxyethane over Noble-Metal-Loaded TiO2 Nanotubes and Nanorods. *ChemSusChem* **2015**, *8* (7), 1226-1231. DOI: https://doi.org/10.1002/cssc.201403305
(16) Zhang, H.; Zhang, W.; Zhao, M.; Yang, P.; Zhu, Z. A site-holding effect of TiO2 surface hydroxyl in the photocatalytic direct synthesis of 1,1-diethoxyethane from ethanol. *Chem. Commun.* **2017**, *53* (9), 1518-1521, 10.1039/C6CC09050H.
